# Do traits of plant species predict the efficacy of species distribution models for finding new occurrences?

**DOI:** 10.1002/ece3.6254

**Published:** 2020-05-12

**Authors:** Jenny L. McCune, Hanna Rosner‐Katz, Joseph R. Bennett, Richard Schuster, Heather M. Kharouba

**Affiliations:** ^1^ Geomatics and Landscape Ecology Research Laboratory Department of Biology Carleton University Ottawa ON Canada; ^2^ Department of Biology University of Ottawa Ottawa ON Canada; ^3^ Present address: Department of Biological Sciences University of Lethbridge Lethbridge AB Canada

**Keywords:** dispersal, generalist, lifespan, niche models, range size, specialist

## Abstract

Species distribution models (SDMs) are used to test ecological theory and to direct targeted surveys for species of conservation concern. Several studies have tested for an influence of species traits on the predictive accuracy of SDMs. However, most used the same set of environmental predictors for all species and/or did not use truly independent data to test SDM accuracy. We built eight SDMs for each of 24 plant species of conservation concern, varying the environmental predictors included in each SDM version. We then measured the accuracy of each SDM using independent presence and absence data to calculate area under the receiver operating characteristic curve (AUC) and true positive rate (TPR). We used generalized linear mixed models to test for a relationship between species traits and SDM accuracy, while accounting for variation in SDM performance that might be introduced by different predictor sets. All traits affected one or both SDM accuracy measures. Species with lighter seeds, animal‐dispersed seeds, and a higher density of occurrences had higher AUC and TPR than other species, all else being equal. Long‐lived woody species had higher AUC than herbaceous species, but lower TPR. These results support the hypothesis that the strength of species–environment correlations is affected by characteristics of species or their geographic distributions. However, because each species has multiple traits, and because AUC and TPR can be affected differently, there is no straightforward way to determine a priori which species will yield useful SDMs based on their traits. Most species yielded at least one useful SDM. Therefore, it is worthwhile to build and test SDMs for the purpose of finding new populations of plant species of conservation concern, regardless of these species’ traits.

## Graphical abstract

We used independent field survey data to assess the accuracy of species distribution models (SDMs) built for 24 plant species of conservation concern. We tested whether traits of species (e.g., dispersal mechanism) or characteristics of their distribution (e.g., mean latitude) influenced model accuracy. Traits do influence species–environment relationships and therefore SDM accuracy, nevertheless a useful SDM could be built for most species.

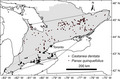

## 1 INTRODUCTION

Species distribution models **(**SDMs) use known locations of a species along with geospatial data on climatic, topographic, edaphic, and land cover variables to predict habitat suitability or probability of occurrence across a region (Guisan & Zimmerman, 2000). SDMs have been used to test ecological theories about what factors constrain species’ ranges (e.g., Kharouba, McCune, Thuiller, & Huntley, 2013; Moore & Elmendorf, 2006), to predict future shifts in species distributions with climate change (e.g., Elith, Kearney, & Phillips, 2010), and in conservation‐related applications (Franklin, 2013; Guisan et al., 2013). For example, SDMs can predict areas of suitable habitat where field surveys might reveal previously undiscovered populations of species of conservation concern (Guisan et al., 2006). Although there are challenges and limitations when using SDMs for this purpose (e.g., Breiner, Guisan, Bergamini, & Nobis, 2015; McCune, 2019), many studies using SDMs to target surveys for plant species have been successful, with SDM‐directed surveys leading to the discovery of previously unknown occurrences—even of species with very few known populations (e.g., Boetsch, Manen, & Clark, 2003; Bourg, McShea, & Gill, 2005; Engler, Guisan, & Rechsteiner, 2004; Guisan et al., 2006; van Manen, Young, Thatcher, Cass, & Ulrey, 2005; Marage, Garraud, & Rameau, 2008; McCune, 2016; Williams et al., 2009). SDMs can effectively complement expert knowledge of the best locations to search, or provide guidance when expert knowledge is lacking. SDM‐directed surveys have been shown to increase the efficiency of field surveys when compared to other sampling strategies (Guisan et al., 2006; van Manen et al., 2005; Rosner‐Katz et al. in revision ). Knowing the locations of all extant occurrences of species of conservation concern is important in order to correctly assess their status and to design effective strategies for recovery.

Although SDMs have had many successful applications, the accuracy of SDMs in representing a species’ geographic distribution varies (e.g., Kharouba et al., 2013). Species with certain traits, which we define broadly as biological characteristics of the species *or* characteristics of a species’ geospatial distribution, might be less amenable to accurate SDMs. For example, many studies have found that SDMs of generalist species tend to be less accurate than SDMs of more specialized species (e.g., Franklin, 2010; Hernandez, Graham, Master, & Albert, 2006; Marshall et al., 2015; Seoane, Carrascal, Alonso, & Palomino, 2005). Presumably, SDMs can more effectively tease apart suitable from unsuitable habitat when the range of conditions the species can tolerate is quite narrow and of limited extent in the study region (Franklin, Wejnert, Hathaway, Rochester, & Fisher, 2009). Dispersal ability may also influence the accuracy of SDMs if poor dispersers are unable to reach all suitable habitat within a study area and are thus absent even where conditions are predicted to be suitable (e.g., Gogol‐Prokurat, 2011; Graham, Silva, & Velásquez‐Tibatá, 2010). Other potentially important traits include lifespan, prevalence in the study area, and range extent (see Table 1).

**Table 1:** Characteristics of plant species or their geographic distributions (“traits”) hypothesized or shown to affect SDM accuracy. Starred traits were retained as candidate predictors after removing highly collinear variables

Trait	Type	Description	Hypothesized influence	References
Woodiness*	Lifespan	Woody or not woody. Woody plants tend to be longer lived	Longer‐lived species have more accurate SDMs because they are more likely to persist long‐term in suitable habitat, whereas herbaceous species are more likely to respond to transient habitat features (e.g., light gaps) which are not easy to model on the scale of SDMs; woody species are more conspicuous and less likely to be overlooked, leading to more accurate presence/absence data	Syphard & Franklin, (2010) Hanspach et al., (2010)
Dispersal type*	Dispersal‐related	Mechanism for seed dispersal. None = no known mechanism (gravity) Animals = seeds dispersed by mammals or birds Wind/none = very tiny seeds that may possibly float on air currents, but may not go far in low‐wind conditions in forest understories Winged = seeds have morphological adaptations for wind dispersal	Species with adaptations for long‐distance seed dispersal are better able to reach all suitable habitat, leading to more accurate SDMs; alternatively, species with shorter dispersal distances may have more accurate SDMs due to adaptations for survival in place rather than dispersal	Graham et al., (2010) Hanspach et al., (2010) Pulliam, (2000) Syphard & Franklin, (2010)
Seed weight*	Dispersal‐related	The average total weight (in grams) of 1,000 seeds/spores. Compiled mainly from the Kew Seed Information Database (Royal Botanic Gardens Kew[, 2019]). Fern spore weight estimated based on largest spore weight recorded in Gómez‐Noguez, Pérez‐Garcia, Mehltreter, Orozco‐Segovia, and Rosas‐Pérez, (2016)	Species with lighter propagules have more accurate SDMs because they can travel farther on wind currents leading to greater ability to reach all suitable habitat (but see above)	
Soil type diversity*	Degree of edaphic specialization	Simpson's diversity index calculated for the soil texture type at all mapped occurrences. Soil texture categorized into 24 types according to the Soil Survey Complex of the Ontario Ministry of Agriculture (see Table 3). Low values indicate greater specialization for soil type.	More specialized species have more accurate SDMs because their range of suitable habitat is limited and easier to differentiate compared with generalists	Brotons et al., (2004) Franklin, (2010) Hernandez et al., (2006) McPherson and Jetz, (2007) Seoane et al., (2005 **)**
Geological diversity		Simpson's diversity index calculated for the surficial geology type at all mapped occurrences. Surficial geology categorized into 40 types according to the Ontario Geological Survey (2010).		
Mean latitude*	Geographic distribution	Mean latitude of all occurrences	Species with more southerly distributions have more accurate SDMs because they are restricted to a small subset of climatic conditions within the study area	Luoto et al., (2005)
Maximum range extent*	Geographic distribution	The largest distance between two occurrences (km) in the study region	Widespread species have less accurate SDMs because local adaptation that varies across the range introduces prediction error	Hernandez et al., (2006) McPherson and Jetz, (2007) Stockwell and Peterson, (2002) Syphard & Franklin, (2010)
Range area	Geographic distribution	The total area of a convex hull enclosing all known occurrences in the study region (ha)		
Total number of occurrences	Prevalence	The total number of occurrences known in the study area	Species with lower prevalence in the study area or sparser records within the species’ range have more accurate SDMs (but the response may be nonlinear)	Luoto et al., (2005) McPherson, Jetz & Rogers,(2004) Tessarolo et al., (2014)
Occurrence density*	Prevalence	The total number of occurrences divided by the species’ range within the study area		

Studies testing the effects of species traits on SDM accuracy have had variable results. Many have found that certain traits do correlate with SDM accuracy (e.g., Franklin et al., 2009; Guisan et al., 2007; Hanspach, Kuhn, Pompe, & Klotz, 2010; Newbold et al., 2010; Poyry, Luoto, Heikkinen, & Saarinen, 2008; Syphard & Franklin, 2009)—but which particular traits are significant varies in studies of different taxonomic groups, and from different regions. Others have found trait‐SDM accuracy correlations lacking or weak (Elith & Burgman, 2002; McPherson & Jetz, 2007; Tessarolo, Rangel, Araujo, & Hortal, 2014). Of course, these tests are complicated by the fact that each species has multiple traits, and a species with one trait that predisposes it to a more accurate SDM might have another trait that acts in the opposite direction. For example, a species might be a good disperser, leading potentially to a more accurate SDM, but also be a generalist, which might make it more difficult to achieve an accurate SDM. This makes it necessary to test the effects of multiple traits simultaneously, so that the effect of each can be tested while accounting for the others. 

In addition, the choice of which environmental predictors to include could affect the accuracy of SDMs, in ways that depend on species traits. For example, two studies found that faster‐growing, disturbance‐associated plants had less accurate SDMs on average than other plant species (Guisan et al., 2007; Hanspach et al., 2010). However, both studies speculate that accuracy would have been higher for those species had geospatial data on the degree of local disturbance been available and included in the SDMs. That is, the distributions of disturbance‐associated species may not be fundamentally difficult to model, as long as the essential predictors are included. A more rigorous test of the effect of species traits on SDM accuracy would account for this by building multiple SDMs for each species that use different sets of environmental predictors.

Another factor to consider when assessing SDM accuracy is the source of the data with which the SDM is evaluated. Often, SDMs are evaluated using a subset of presences and absences withheld from the same dataset used to build the model (i.e., not truly independent data). Many studies evaluating the effect of traits on SDM accuracy have used this method (e.g., Hernandez et al., 2006, McPherson & Jetz, 2007, Poyry et al., 2008, Syphard & Franklin, 2010, Tessarolo et al., 2014). However, SDM accuracy tends to be higher using this technique compared with when independently collected presence and absence data are used (Elith & Burgman, 2002; McCune, 2016, Newbold 2010). This is probably because environmental or spatial biases in the full set of occurrences available for SDM building are retained in the subset withheld for evaluation (Chatfield, 1995). For targeting suitable habitat for field surveys, it is crucial that the SDM is able to predict habitat suitability accurately at new sites. Therefore, when testing the potential effects of species traits on SDM accuracy, it is important to assess SDMs using independently collected presence and absence data whenever possible.

In this study, we ask whether traits of species or their geographic distributions affect the accuracy of SDMs for 24 plant species of conservation concern. Our goal was to test the fundamental question: “are species’ traits related to the strength of the relationship between environmental predictors and geographic distribution?” and the applied question that follows from it: “can traits be used to predict which species will be most amenable to the use of SDMs to target field surveys?” We built eight SDM versions for each species, varying the set of environmental predictors included in each version to account for the potential for the accuracy of an SDM to vary depending on the predictors, and recognizing that the most accurate SDM version might be different for different species. Specifically, we included land cover and forest extent in some SDM versions based on our observation in a previous study that some species respond to forest type and landscape context in addition to climatic, topographic, and edaphic conditions (McCune, 2016). We then evaluated the SDMs with independent presences and absences obtained from field surveys of 156 sites that varied in their predicted habitat suitability for each species. Thus, our measure of SDM accuracy measures the ability of the SDMs to predict habitat suitability at new sites.

## 2 METHODS

### 2.1 Study area and species

**Figure 1:**
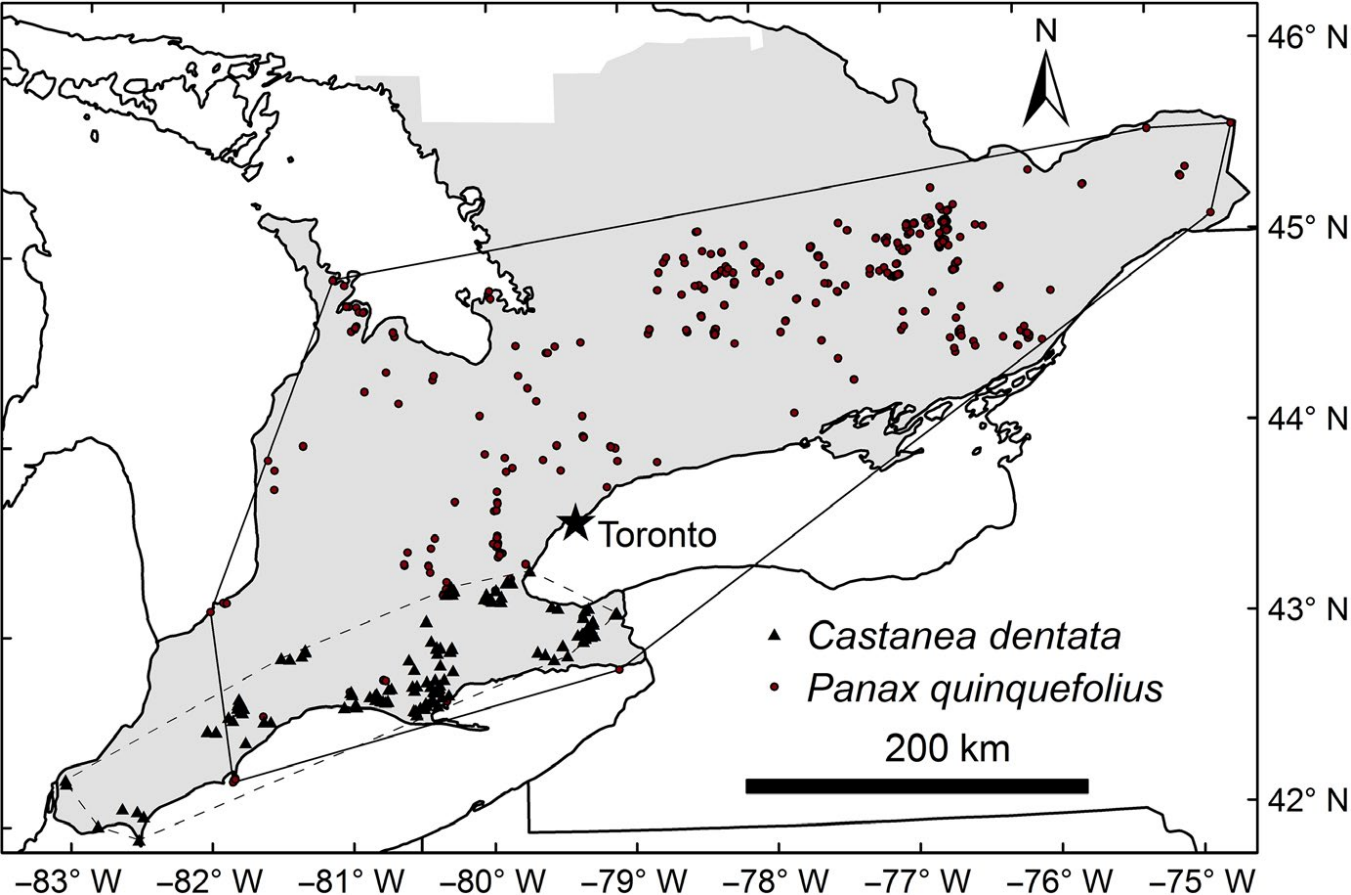
The study area (shaded) in southern Ontario. Occurrence records used to build SDMs for *Panax quinquefolius* (wild ginseng, circles) and *Castanea dentata* (American chestnut, triangles) are shown as examples. Polygons are convex hulls enclosing the extent of the records of each species in the study area

Our study region is southern Ontario, Canada (Figure 1), in the Mixedwood Plains ecozone (Crins, Gray, Uhlig, & Wester, 2009). This is the most highly populated region of the province. Prior to European colonization, the region was dominated by forest, but forests are now highly fragmented and the landscape is dominated by agriculture (Larson, Riley, Snell, & Godschalk, 1999). Mean daily temperatures range from 18 to 22°C in July, and there is 720–1,000 mm of annual precipitation (Crins et al., 2009). Elevation ranges from 20 to 575 m above sea level.

Southern Ontario is a hotspot for plant diversity in Canada: More than 40% of Canada's plant species occur here (Oldham, 2017). The region is also home to many threatened plant species: 80 out of 201 vascular plants listed as extirpated, endangered, threatened, or special concern under Canada's Species at Risk Act occur in Ontario (Government of Canada, 2018). We built SDMs for 41 vascular plant species that are provincially rare (ranked S1, S2, or S3 in Ontario, indicating critically imperiled, imperiled, or vulnerable status, respectively; Faber‐Langendoen et al., 2012; Table 2). To maximize the accuracy of field surveys, we chose species that are relatively easy to identify in the field. We also focused on those species that grow mainly in woodland habitats. The species vary in their habitat specificity, their longevity, the number of known occurrences in Ontario, and the extent of their distribution within the study region (Table 2). Most of these species are at the northern edge of their range in southern Ontario, which extends into the southeastern United States. We compiled data on traits of each species that we hypothesized might influence the accuracy of SDMs, based on previous studies (Table 1).

**Table 2:** Plant species for which we built SDMs, and values for each tested trait. For explanation of traits, see Table 1. Only species for which we obtained at least 10 independent presence records are shown

Species	Family	Woodiness	Dispersal type	Seed weight (grams/1,000 seeds)	Soil type diversity (lower = more specialized)	Mean latitude (UTM 17N)	Maximum range extent (km)	Occurrence density (occurrences per 100 square km)
*Aplectrum hyemale*	Orchidaceae	Not woody	Wind/none	0.0015	0.5391	4,823,988	332	0.08
*Arisaema dracontium*	Araceae	Not woody	Animal	50.1150	0.5919	4,763,055	354	0.59
*Asimina triloba*	Annonaceae	Woody	Animal	847.0000	0.6914	4,723,402	342	0.35
*Asplenium scolopendrium*	Aspleniaceae	Not woody	Wind/none	0.0001	0.5585	4,930,190	215	2.62
*Castanea dentata*	Fagaceae	Woody	Animal	3,467.3000	0.6024	4,744,171	340	2.28
*Celtis tenuifolia*	Ulmaceae	Woody	Animal	93.6544	0.4772	4,754,197	532	0.32
*Cornus florida*	Cornaceae	Woody	Animal	102.0000	0.6338	4,761,902	348	1.43
*Cypripedium arietinum*	Orchidaceae	Not woody	Wind/none	0.0019	0.7490	4,964,400	622	0.12
*Enemion biternatum*	Ranunculaceae	Not woody	None	2.7672	0.3788	4,755,022	70	4.29
*Erigenia bulbosa*	Apiaceae	Not woody	None	2.2570	0.6397	4,756,502	323	0.22
*Eurybia divaricata*	Asteraceae	Not woody	Wind/none	0.6443	0.2364	4,769,268	66	4.27
*Frasera caroliniensis*	Gentianaceae	Not woody	None	9.4498	0.4790	4,782,447	102	1.67
*Fraxinus quadrangulata*	Oleaceae	Woody	Winged	70.1800	0.7258	4,663,494	322	0.99
*Gymnocladus dioicus*	Fabaceae	Woody	None	1843.0000	0.7204	4,658,654	345	0.85
*Heuchera americana*	Saxifragaceae	Not woody	Wind/none	0.0252	0.5692	4,638,165	65	6.81
*Hydrastis canadensis*	Ranunculaceae	Not woody	Animal	10.9036	0.5668	4,732,533	328	0.34
*Juglans cinerea*	Juglandaceae	Woody	Animal	14,026.0000	0.4979	4,955,907	790	1.92
*Liparis liliifolia*	Orchidaceae	Not woody	Wind/none	0.0040	0.4182	4,702,675	595	0.23
*Lithospermum latifolium*	Boraginaceae	Not woody	None	21.7810	0.5518	4,779,621	189	0.30
*Magnolia acuminata*	Magnoliaceae	Woody	Animal	88.5100	0.6343	4,743,870	251	0.37
*Mertensia virginica*	Boraginaceae	Not woody	None	2.9223	0.4152	4,744,859	257	0.27
*Nyssa sylvatica*	Nyssaceae	Woody	Animal	140.0000	0.6526	4,731,319	346	0.38
*Panax quinquefolius*	Araliaceae	Not woody	Animal	27.7540	0.3724	4,941,323	701	0.66
*Phegopteris hexagonoptera*	Thelypteridaceae	Not woody	Wind/none	0.0001	0.5329	4,790,511	636	0.12

SDM, species distribution models.

### 2.2 Species distribution models

We obtained georeferenced data on known occurrences of each species in southern Ontario from the Natural Heritage Information Centre (NHIC) of the Ontario Ministry of Natural Resources and Forestry (OMNRF). The NHIC maintains records of all species ranked S1, S2, or S3 from herbarium records and confirmed sightings by provincial biologists, other scientists, or members of the public (Government of Ontario, 2018). We chose a resolution of 100 × 100 m (1 hectare) for the SDMs, corresponding to the largest area we felt could be comprehensively surveyed for rare plants within a reasonable amount of time. We therefore removed all occurrence records with a spatial uncertainty greater than 100 m.

We compiled geospatial data on climate, soils, surficial geology, and topography from publicly available spatial datasets and converted them to a 100 × 100 m resolution (Table S1). Given the large number of climatic variables available, we chose a set that were minimally correlated (*r* < 0.7). Our primary set of predictors included 14 variables representing topography, surficial geology, soils, and climate only. SDMs for some species (but not all) are more accurate for sites with more contiguous forest surrounding them (McCune, 2016). Therefore, we used the Southern Ontario Land Information System (SOLRIS; Smyth, 2008) wooded layer, which delineates all forested areas in southern Ontario, to calculate the number of cells within the 9 × 9 cell area surrounding each focal cell that were forested. We called this “forest contiguity.” A 9 × 9 cell area corresponds to approximately the same area as is encompassed by a circular area with a 500 m buffer around each focal cell, which is a distance at which correlations between forest amount and presence or absence of plant species of conservation concern has been shown (McCune, 2016; McCune, Natto, & MacDougall, 2017). We also used the SOLRIS categorical representation of land cover as a predictor in some of the SDMs (Table S1).

We used the program MaxEnt to build 8 SDMs for each of the 41 species (Phillips, Anderson, & Schapire, 2006; we refer to the 8 SDMs for each species as “SDM versions”). We chose MaxEnt because it performs as well or better than other SDM methods, especially for presence‐only data and in cases where the number of presence records is low (Hernandez et al., 2006; Williams et al., 2009). In addition, the pilot study showed that MaxEnt could produce accurate predictions of habitat suitability for plant species in our region, when assessed with independent presence and absence records (McCune, 2016). Because we had evidence of its efficacy based on independent data, we chose to use MaxEnt alone rather than using multiple SDM techniques and creating ensemble SDMs. While some advocate including all possible predictors and allowing MaxEnt to hone in on the most important (e.g., Phillips et al., 2006), we have found that SDMs built with a smaller subset of potential variables were more accurate for some species. Therefore, we used 4 sets of environmental predictors for each species: (a) the original 14 (climate, topography, soil, and geology only), (b) the original 14 plus forest contiguity, (c) the original 14 plus land cover, or (d) the original 14 plus forest contiguity and land cover (Table S1).

We built two SDMs with each of these four predictor sets, by varying the regularization multiplier in MaxEnt. The first model used the default value of 1, while the second used a value of 0.5. During initial trials building SDMs, we experimented with setting the regularization multiplier at 0.5, 1, or 3 and found that the first two always led to better performing SDMs. Regularization is a method used by MaxEnt to penalize models that are too complex, reducing overfitting (Elith et al., 2011; Merow, Smith, & Silander, 2013). By changing the regularization multiplier to 0.5, we reduced the strength of this penalization. Varying the regularization multiplier is recommended by Merow et al. (2013). We set the background for pseudo‐absences in MaxEnt as the entire study area (shaded area in Figure 1).

We tested the accuracy of each SDM using independent presences and absences from detailed forest surveys we carried out in 2014 and 2015, between May and September of each year (McCune et al., 2017). We surveyed 156 100 × 100 m cells that varied in their predicted habitat suitability for each modeled species, as defined by MaxEnt's cumulative output for each cell. This value varies from 0 to 100, with values closer to 100 indicating higher predicted habitat suitability. We chose sites with the objective of maximizing the range of predicted habitat suitability for each species, while including at least ten high‐suitability sites for each species. We were also limited to sites for which we could obtain landowner permission to access. Given the variation in habitat associations of the species, the 156 sites encompassed a wide range of predicted habitat suitability for each species, with the lower end of the range especially well represented. Our crew of 2–4 field technicians navigated to the center of each cell using a handheld GPS unit and then flagged 50 m in each cardinal direction using a rangefinder and compass to delineate four quadrants. We then systematically searched each quadrant by walking transects no more than 5 m apart and recording all vascular plant species present. Survey times ranged from 5 to 10 person hours. We considered a cell in which a species was not found to be a true absence only if we surveyed the cell during the time of year when the species was likely to be present and recognizable (e.g., for spring ephemerals, we did not use any apparent absences from plots surveyed later than mid‐June).

Although we found new occurrences of 15 of the 41 species, we did not find more than eight new occurrences for any species. Therefore, we obtained additional independent presence records from the NHIC. These were submissions of sightings to the NHIC that had not yet been incorporated into the main NHIC database. We ensured that these records were spatially accurate and did not occur in the same cell as any of the records used to build the SDMs. We selected those species which had at least 10 independent presence records, including those from our surveys and these extra independent presence records combined. This resulted in a final list of 24 species (Table 2).

We used the independent presence and absence records to calculate the AUC (area under the receiver operating characteristic curve) and the TPR (true positive rate) for each of the eight SDMs for each species. The AUC is a threshold‐independent index that measures the ability of an SDM to correctly discriminate between presences and absences. It ranges between 0 and 1, where values of 0.5 or less indicate that the SDM is worse than a random model would be at predicting presence or absence (Fielding & Bell, 1997). The TPR is the proportion of independent presence records that were predicted to have suitable habitat by the SDM. We set the threshold for “suitable” habitat as the minimum MaxEnt output value that resulted in the correct prediction of 90% of the presence records used to build the model, as long as there were at least 15 original records. If there were fewer than 15 records used to build the SDM, we set the threshold at the MaxEnt output value that resulted in the correct prediction of 100% of the original presence records, as recommended by Pearson, Raxworthy, Nakamura, and Peterson (2007). TPR is an important metric when evaluating SDMs for use in targeting surveys for species because high rates of omission errors would result in many occupied sites not being surveyed due to the SDM incorrectly predicting them to be unsuitable. Therefore, the most useful SDMs for directing field surveys must do a good job of correctly predicting occurrences. The AUC and TPR for each species and each SDM version are available in the Supplementary Material (Tables S3 and S4).

### 2.3 Testing the relationship between SDM accuracy and species traits

We began with 10 traits, 8 continuous and 2 categorical (Table 1). Prior to the analysis, we assessed multicollinearity among continuous candidate predictors using variance inflation factors (VIF; Zuur, Ieno, & Elphick, 2010). We also calculated pairwise Pearson's correlation coefficients (Table S2). We used the vifstep function (“usdm” package in R) to choose a set of relatively independent variables with a VIF less than 3 (*n* = 7; Table 1). We log‐transformed seed weight and density of occurrence to improve normality. This resulted in a set of seven candidate predictors: maximum range extent, density of occurrence, soil type diversity, mean latitude, seed weight, seed dispersal type (categorical), and woodiness (categorical; see Table 2). We standardized all continuous variables before building models.

We analyzed each of the two accuracy measures separately. Because AUC is a continuous proportion that ranges from 0 to 1, we used a generalized linear mixed effects model (glmm) with a beta error distribution (“glmmTMB” function). We included SDM model version and regularization factor as random effects to account for potential differences in model performance that might be introduced by SDMs built using different predictors or with different regularization settings. Due to nonlinearity, we fit seed weight as quadratic. We first fit a model including all seven candidate predictor variables. We then used the dredge function of the MuMIn package to create and rank a list of all possible variable combinations based on the lowest AICc. We assessed the relative importance of each of the variables retained in the top ranked model by using a drop1 test to assess the importance of each predictor once all the others were accounted for, based on the difference in AICc with and without the predictor. We report both dAICc and the p‐value of χ^2^ tests for each comparison. We assessed overall fit of the top ranked model using an R^2^ function specified for the beta distribution based on Nakagawa, Johnson, and Schielzeth (2017) and report the conditional value.

Because TPR is a continuous computed rate based on the number of correctly predicted presences (“successes”) out of the total number of independent presences (“trials”), we analyzed this response variable using a glmm with a binomial error distribution (“glmmTMB” function), including SDM model type and regularization factor as random effects. We assessed model fit and variable importance using the same procedure described above. We assessed overall model fit using R^2^ (“r.squaredGLMM” function).

We chose to use the drop1 test on the top ranked model to assess the relative importance of each predictor rather than taking a model averaging approach and calculating relative importance values (Burnham & Anderson, 2002) because the averaging of partial regression coefficients across multiple models is not recommended when dealing with predictor variables that are not completely orthogonal or for models with a nonlinear link function, as is the case here (Banner & Higgs, 2017; Cade, 2015).

We used partial residual plots to visualize the final model for each response variable. These plots show the predicted effect of each trait variable in the final model, while holding the other predictors constant at their median or the most frequent category (visreg package; Breheny & Burchett, 2016). We conducted all statistical analyses in R version 3.5.1.

## 3 RESULTS

Across all species and all SDM versions, AUC (as calculated with independent data) ranged from 0.36 to 0.98, while TPR ranged from 0 to 0.96 (Table S4). The most accurate SDM varied according to species: For some species, SDM accuracy increased when land cover and/or forest contiguity were included, while for others, the SDM with the highest AUC or TPR included only climatic and topographic predictors (Figures S1 and S2). The range of variability between SDM versions also varied. For example, AUC for *Liparis lillifolia* ranged from 0.96 to 0.97, depending on SDM version, while AUC for *Aplectrum hyemale* ranged from 0.59 to 0.73.

**Figure 2:**
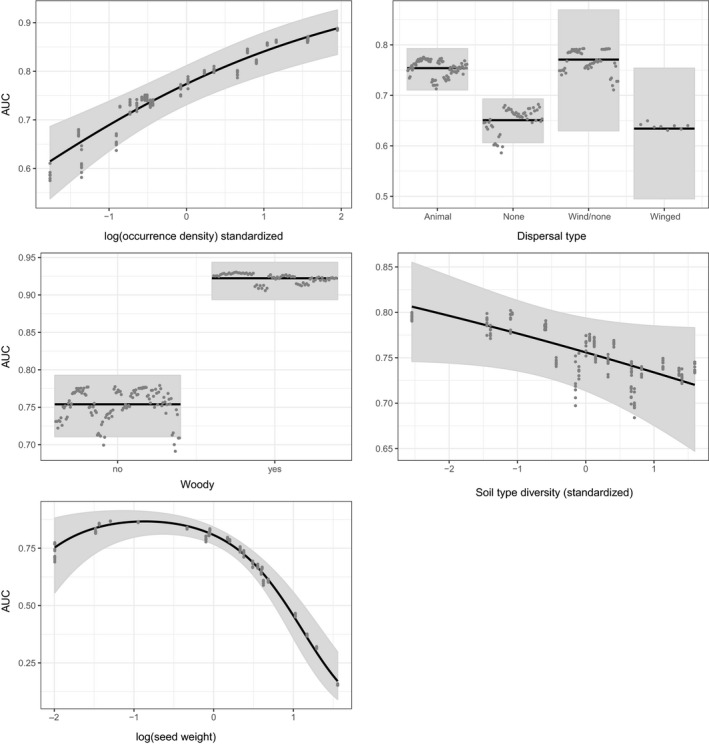
Partial residual plots showing the effect of each variable in the final model on the AUC (area under the receiver operating characteristic curve) as determined by independent field surveys. Note that for each variable, all other variables are held constant at their median (or the most common category, for categorical variables)

The best model for predicting AUC of SDMs included five predictor variables: occurrence density, woodiness, seed weight, dispersal type, and soil type diversity (Table 3a). The best model had an R^2^ value of 0.88. The most influential variable was occurrence density—dropping it from the model led to an increase in AICc of 50.3 (Table 3a). All other predictors being equal, species with a higher density of occurrences within their range in the study area, woody species, species with smaller seeds, and species with animal‐ or likely wind‐dispersed seeds had higher AUC than others (Figure 2).

**Table 3:** The relative importance of species’ traits predicting (a) AUC and (b) TPR. Model terms are listed in order of decreasing influence as measured by the difference in AICc between a model without the variable and the full model

Model	Estimate (SE)	*df*	dAICc	Chisq	*p*‐value
(a) AUC					
Full		12	0		
Occurrence density	0.45 (0.06)	11	50.3	52.58	<.001
Woodiness	1.38 (0.19)	11	44.3	46.56	<.001
Log (seed weight)	−1.06 (0.15)	11	42.4	44.64	<.001
Log (seed weight)^2^	−0.61 (0.07)	11	42.4	44.64	<.001
Dispersal type	NA	9	12.7	19.5	<.001
Soil type diversity	−0.10 (0.06)	11	1.0	3.23	.07
(b) TPR					
Full		12	0		
Dispersal type	NA	9	329.2	336.0	<.0001
Woodiness	−1.27 (0.1)	11	153.3	155.6	<.0001
Occurrence density	0.63 (0.05)	11	145.9	148.2	<.0001
Latitude	−0.46 (0.04)	11	121.4	123.7	<.0001
Log (seed weight)	−0.48 (0.1)	11	22.0	24.3	<.0001
Maximum range extent	0.22 (0.06)	11	13.2	15.5	<.0001
Soil type diversity	0.10 (0.04)	11	3.1	5.4	.02

AUC, area under the receiver operating characteristic curve; TPR, true positive rate.

**Figure 3:**
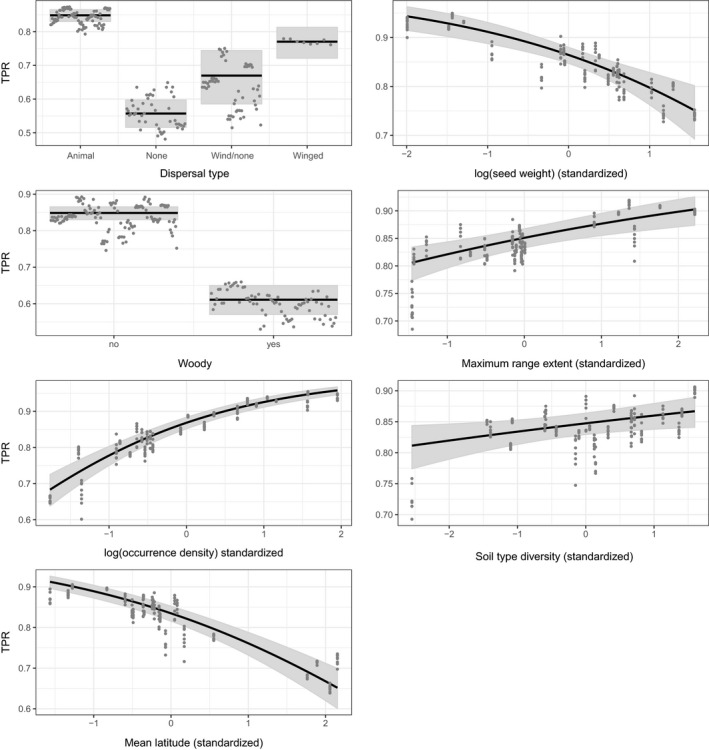
Partial residual plots showing the effect of each variable in the final model on the TPR (true positive rate) as determined by independent field surveys. Note that for each variable, all other variables are held constant at their median (or the most common category, for categorical variables)

The final model for TPR included seven predictors: dispersal type, density of occurrences, maximum range extent, seed weight, latitude, soil specialization, and woodiness (Table 3b). The final model had an R^2^ value of 0.17. Dispersal type and woodiness contributed most to the model individually. All else being equal, species with winged seeds or seeds dispersed by animals, and nonwoody species had higher TPR (Figure 3). The other five predictors also had significant effects, with species having a higher density of occurrences within their range, lower mean latitude, lighter seeds, larger range extents, and growing on a greater diversity of soil types having higher TPR (Figure 3).

Some patterns were common to both response variables: Species with animal‐dispersed seeds, lighter seeds, and a greater density of occurrences had higher AUC and TPR, all else being equal. However, woodiness showed opposing effects, with woody species having higher AUC but lower TPR. Edaphic specialization, as measured by the diversity of soil types occupied, had a marginal effect on both AUC and TPR, but with opposite effects: AUC declined with increasing diversity of soil types occupied, while TPR increased (Table 3). Mean latitude had an effect on TPR only, which was negative (Table 3).

## 4 DISCUSSION

Our results support the idea that the traits of species can influence the strength of the relationship between environmental predictors and species’ occurrences, and hence the accuracy of SDMs. Characteristics of the species’ life history and geographic distribution significantly influenced the overall accuracy of the SDMs and the true positive rates of independent surveys. Most traits influenced SDM accuracy as predicted by theory and confirmed in previous studies (Table 1). However, some traits influenced only AUC or TPR, not both, and the effect of traits on TPR was opposite to the predicted effect for four of the seven traits.

### 4.1 Lifespan

SDMs for woody species, which tend to be longer lived than herbaceous plants, had higher AUC values, all other traits held constant. Syphard and Franklin (2010) and Hanspach et al. (2010) also found that longer‐lived plants tended to produce SDMs with greater AUC. In contrast, woody species had significantly lower TPR. This suggests that SDMs for woody species have higher accuracy when considering both types of error (false negatives and false positives), but do a poorer job of correctly predicting independent presences of woody species compared with herbaceous species. The higher rate of omission for woody species could indicate the greater ability of long‐lived species to tolerate habitat that has recently become less suitable. However, this hypothesis would only apply for SDMs built using predictors that have changed substantially over recent decades, most notably forest contiguity.

### 4.2 Dispersal‐related traits

Species dispersed by mammals or birds tended to have higher AUC than species with no long‐distance dispersal mechanism, consistent with the idea that species able to disperse their seeds farther are better able to colonize suitable habitat throughout the study region. SDMs for mammal or bird dispersed species also tended to have higher TPR, indicating that they are less prone to false negatives. Species with no evident specialized dispersal mechanism had the lowest TPR and lower AUC than animal or wind‐dispersed species. Syphard and Franklin (2010) found that plants with ballistic dispersal had higher AUC than others, with animal and wind‐dispersed species having slightly lower AUC scores, and gravity dispersed species the lowest.

Species with lighter seeds had significantly more accurate SDMs, all other traits being equal. This is consistent with the idea that species with lighter seeds are able to travel farther (e.g., McEuen & Curran, 2004) and thus have distributions that more closely match environmental conditions. This result was the same for both measures of model accuracy, although the relationship for AUC was nonlinear. Our results for TPR indicate that species with lighter seeds tend to be found in sites that are predicted to be suitable by the SDM more often than species with heavier seeds. Heavier‐seeded species may not be able to track suitable habitat as closely due to their reduced ability to disperse great distances.

### 4.3 Edaphic specialization

We did not find a strong role for edaphic specialization in influencing the relationship between environmental predictors and a species’ distribution. The degree of specialization, as measured by the diversity of soil types occupied, was maintained in the final model for both AUC and TPR, but in both cases was the predictor with the least influence once other predictors had been accounted for. There was a negative relationship between soil type diversity and AUC, indicating that species that are more specialized had more accurate SDMs. This result aligns with Hernandez et al. (2006), who found that AUC increased for animal species with narrower niche tolerance, and Marshall et al. (2015), who found that highly specialized bee species were modeled more accurately than generalists. Similarly Seoane et al. (2005) and Brotons, Thuiller, Araujo, and Hirzel (2004) found that birds with specialized habitat selection had more accurate habitat suitability models. In contrast, Hanspach et al. (2010) did not find an effect of plant species specialization, as measured by the number of different vegetation types with which a species was affiliated. Interestingly, in our study, the relationship was reversed for TPR, with species found on a more diverse range of soil types having higher TPR, all else being equal. We believe that the range of soil types upon which a species is found is a good indication of specialization in plants. However, for species with very few records such as some of the rarest species in our dataset, the calculation of this indicator may be biased due to low numbers of extant populations.

### 4.4 Geographic distribution

We detected an effect of average latitude on SDM performance for TPR only. Luoto, Poyry, Heikkinen, and Saarinen (2005) built SDMs for 98 butterfly species in Finland and found that butterflies at the margin of their geographic range in Finland (occurring at lower latitudes on average) tended to have more accurate SDMs. They suggested that these range edge species are likely restricted to a narrower range of habitats within Finland, thus making them more amenable to accurate SDMs. This is likely also true for the plant species in our study, as those with smaller mean latitude tend to be restricted to the Carolinian forest zone, which reaches the northernmost extent of its range in the far southwest of southern Ontario and is therefore limited in area. Hence, SDMs for species like American chestnut (*Castanea dentata*), which are restricted to the Carolinian zone, may yield more accurate SDMs than species like wild ginseng (*Panax quinquefolia*), which range widely throughout our study area (Figure 1). However, it is worth noting that the TPR for these two species was practically equivalent, which highlights the importance of other traits that can influence SDM accuracy. In addition, we did not find an effect of mean latitude on AUC.

Range size within the study region was not a significant predictor of AUC. This contrasts with the findings of Hernandez et al. (2006), McPherson and Jetz (2007), and Syphard and Franklin (2010), all of whom found a negative correlation between range size and SDM accuracy. Syphard and Franklin (2010) speculate that range size is often correlated with the degree of environmental variation (i.e., specialization) of the species, which could explain why species with smaller ranges have higher AUC. However, in our study, the two were not strongly correlated (R^2^ = 0.10, Table S2), and we included both as potential predictors in our models. McPherson and Jetz (2007) suggest that species with larger range sizes may exhibit variation in habitat preferences across the range, making them more difficult to model due to variation in the relationship between environmental predictors and presence/absence across the range. It is possible that within our relatively small study region, no such variation in habitat preferences or local adaptation exists.

We did find an effect of range size on TPR, but in the opposite direction than predicted: Species with larger ranges tended to have a higher TPR. Garrison and Lupo (2002) found that birds with larger range sizes yielded more accurate SDMs. However, McPherson and Jetz (2007) suggest that this could be a result of correlation of larger range size with greater prevalence, with greater prevalence driving increased SDM accuracy. In our study, range size in the study region and occurrence density (our measure of prevalence) were not strongly correlated, so we do not think this is the cause of the increase in TPR with range size.

Finally, both AUC and TPR were positively related to occurrence density. This was opposite to results from other studies (e.g., Luoto et al., 2005; Tessarolo et al., 2014) that found AUC to be negatively related to prevalence. However, it is important to note that these studies define prevalence as the number of occupied grid cells divided by the total number of grid cells in the study area. Our measure of occurrence density, in contrast, accounted for range size by measuring the density of occurrences within the total range extent encompassed by known occurrences within our study region. A greater density of occurrences within the range extent of a species most likely gives a better sample of the range of environmental conditions in which a species can survive, allowing MaxEnt to more accurately differentiate “suitable” versus “unsuitable” conditions, and leading to the higher AUC and TPR for species with higher occurrence density.

## 5 CONCLUSIONS

It is important to note that our results may be linked to the extent and resolution of our study. For example, in a study at a much smaller extent (0.33 km^2^) and finer resolution, Moore and Elmendorf (2006) found that the ability of SDMs to predict plant species distributions was not affected by dispersal mechanism or seed size. Given the scale dependence of most ecological patterns and processes (e.g., Levin, 1992), this is a caveat common to most ecological studies. However, the scale and resolution of our study is comparable to many studies that have used SDMs to target field surveys for plants (e.g., Boetsch et al., 2003; Engler et al., 2004; Gogol‐Prokurat, 2011; Marage et al., 2008; Williams et al., 2009).

We found significant effects of species traits on SDM performance, which supports the idea that species traits are related to the strength of the relationship between environmental predictors and geographic distribution. However, the answer to our second question—“can traits be used to predict which species will be most amenable to the use of SDMs to target field surveys?”—is not straightforward. First, because each species has multiple traits, it would be difficult to predict a priori for which species it will be possible or impossible to build a useful SDM. For example, although woody species tended to have higher AUC, the species in our dataset with the highest AUC were *Asplenium scolopendrium* (a fern) and *Liparis liliifolia* (an orchid; Table 2). Second, some traits affected TPR in the opposite direction to our predictions and/or in the opposite direction than they affected AUC. Species with traits that tend to decrease AUC may nonetheless yield SDMs with excellent TPR, which is perhaps more important when using SDMs to target rare plant surveys. Third, there was a great deal of variation in TPR that was unexplained by traits (R^2^ = 0.17), so any prediction for a particular species based on our glmm might not reflect the true ability of an SDM to predict independent occurrences. This matches the results of McPherson and Jetz (2007), who found that bird species traits explained only about 20% of the variation in SDM performance. Finally, although there was variation in SDM performance, we were able to build at least one “useful” SDM for most species. Twenty out of 24 species had at least one SDM version with AUC >0.7, and 17 out of 24 species had at least one SDM version with TPR >70%. Therefore, it is worthwhile to build and test SDMs for the purpose of finding new populations of plant species of conservation concern, regardless of their traits. We recommend building different SDM versions using a range of environmental predictors to help ensure the best SDM possible and using independently collected presence and absence data to test SDM accuracy.

## CONFLICT OF INTEREST

The authors declare no conflict of interest.

## AUTHOR CONTRIBUTIONS

**Jenny L. McCune:** Conceptualization (lead); Data curation (lead); Formal analysis (supporting); Funding acquisition (lead); Investigation (lead); Methodology (lead); Project administration (lead); Writing‐original draft (lead); Writing‐review & editing (lead). **Hanna Rosner‐Katz:** Data curation (supporting); Formal analysis (supporting); Writing‐review & editing (supporting). **Joseph R. Bennett:** Funding acquisition (supporting); Project administration (supporting); Supervision (supporting); Writing‐review & editing (supporting). **Richard Schuster:** Formal analysis (supporting); Writing‐review & editing (supporting). **Heather M. Kharouba:** Formal analysis (lead); Writing‐review & editing (supporting).

## Supporting information

Table S1

## Data Availability

Climate data and MaxEnt input files freely available online, see Supporting Information Table S3. Rare plant species occurrence data can be obtained from the Ontario Natural Heritage Information Centre upon request and completion of Data Sensitivity Training. Trait data and SDM accuracy data for all species and all SDM versions available in Supporting Information, see Tables S1 and S2, and Dryad https://doi.org/10.5061/dryad.rv15dv452.
