# Supplementary material for: Do traits of plant species predict the efficacy of species distribution models for finding new occurrences?
Source: Ecol Evol. 2020 May 12;10(11):5001–14. doi: 10.1002/ece3.6254 (PMC7297770; doi:10.1002/ece3.6254)
Supplement: Supplementary file 1 — Table S1 [file ECE3-10-5001-s001.pdf]

Supporting Information for "Do traits of plant species predict the efficacy of species distribution models for finding new occurrences?" by J.L. McCune, Hanna Rosner-Katz, Joseph R. Bennett, Richard Schuster, and Heather M. Kharouba

Table S1: Environmental Predictors used to build Species Distribution Models, and their source (open access)

type	variable	description	source/reference	web
'original' set: topography, soil, geology, climate	elevation	elevation in metres	Canadian Digital Elevation Model	http://geogratis.gc.ca/
	slope	slope in degrees	Canadian Digital Elevation Model	http://geogratis.gc.ca/
	aspect	aspect converted to a linear variable using formula in Williams et al. 2009	Canadian Digital Elevation Model	http://geogratis.gc.ca/
	soil texture	texture of the majority soil type, e.g. "clay loam", categorical with 24 categories	Soil Survey Complex, Ontario Ministry of Agriculture	https://www.ontario.ca/page/land-information-ontario
	soil drainage	drainage of the majority soil type, e.g. "well-drained", categorical with 9 categories	Soil Survey Complex, Ontario Ministry of Agriculture	https://www.ontario.ca/page/land-information-ontario
	surficial geology	main type of surficial deposit, categorical with 40 categories	Surficial Geology of Southern Ontario, Ontario Geological Survey 2010	https://www.ontario.ca/page/land-information-ontario
	annual mean temperature	the average of the average monthly temperature (°C)	Canada Forest Service (McKenney et al. 2011)	https://cfs.nrcan.gc.ca/projects/3/2
	mean temperature of the growing season	average temperature during the growing season (°C)	Canada Forest Service (McKenney et al. 2011)	https://cfs.nrcan.gc.ca/projects/3/2
	isothermality	measure of how large the day-to-night temperature oscillation is in comparison to the summer-to-winter oscillation	Canada Forest Service (McKenney et al. 2011)	https://cfs.nrcan.gc.ca/projects/3/2
	mean temperature of the wettest quarter	the average temperature for the three months with the highest cumulative precipitation (°C)	Canada Forest Service (McKenney et al. 2011)	https://cfs.nrcan.gc.ca/projects/3/2
	annual precipitation	sum of all totally monthly precipitation (mm)	Canada Forest Service (McKenney et al. 2011)	https://cfs.nrcan.gc.ca/projects/3/2
	total precipitation for the growing season	sum of precipitation recorded during growing season (mm)	Canada Forest Service (McKenney et al. 2011)	https://cfs.nrcan.gc.ca/projects/3/2
	precipitation seasonality	variation in monthly precipitation over one year (%)	Canada Forest Service (McKenney et al. 2011)	https://cfs.nrcan.gc.ca/projects/3/2
	precipitation of the warmest quarter	total precipitation for the three warmest months (mm)	Canada Forest Service (McKenney et al. 2011)	https://cfs.nrcan.gc.ca/projects/3/2
land cover type	land use/land cover	land cover type, e.g. "deciduous forest", categorical with 25 categories	Southern Ontario Land Resource Information System (MNRF)	https://www.ontario.ca/data/southern-ontario-land-resource-information-system-solris-20
landscape context	forest contiguity	calculated the number of cells within an 81 cell neighbourhood including the focal cell that are >50% forested	Southern Ontario Land Resource Information System (MNRF)	https://www.ontario.ca/data/southern-ontario-land-resource-information-system-solris-20

Table S2: Pearson Correlation Coefficients for candidate traits. Variables with a Pearson Correlation Coefficient of 0.50 or greater are in bold lettering.

	woodiness	dispersal type	log (seed weight)	soil type diversity	geological diversity	mean latitude	maximum range extent	range area	total # of occurrences	log (occurrence density)
woodiness		0.43	**0.70**	0.47	0.17	0.24	0.19	0.14	0.33	0.10
dispersal type			**0.71**	0.04	0.07	0.07	0.18	0.25	0.32	0.00
log (seed weight)				0.17	0.17	0.23	0.03	0.16	0.41	0.24
soil type diversity					0.42	0.17	0.10	0.00	0.09	0.28
geological diversity						0.21	0.08	0.03	0.08	0.09
mean latitude							0.50	**0.70**	0.50	0.11
maximum range extent								**0.86**	**0.55**	0.47
range area									**0.77**	0.20
total # of occurrences										0.25

Table S3: explanation of columns in Table S4

FIELD	description
species	plant species
family	family
model	specification of the model: A = original (14 original climatic/topographic/edaphic predictors, except HEUCAME which excluded climate), B = original plus land use/land cover data, C = original plus forest contiguity, D = original + land use/land cover + forest contiguity
reg	The regularization setting in MaxEnt. A = 1, B = 0.5
obs	# of observations (unique per 1 ha cell) used to build the SDM
abs_indep	# of independent ABSENCE records used to test the SDM (From field surveys.)
pres_indep	# of independent PRESENCE records used to test the SDM (*** all independent from the records used to build the model. From field surveys + additional data records held by the province or other researchers)
pres_survey	# of new occurrences discovered during field surveys in 2014 and 2015
totpres	total number of presences (obs + independent data) unique per 1 ha cell (may be greater than obs + pres_indep due to "NAs" for some environmental predictors preventing the use of some of these presences by MaxEnt)
AUC_test	the AUC provided by MaxEnt, calculated using 25% of the occurrence records withheld from the training data, averaged over 10 runs
AUC_indep	AUC based on the independent presences and absences
TPR_mpa	True positive rate based on the independent presences - with threshold for presence set at the number that predicts 90% of occurrences used to build the model to be present (if obs is 15 or more) or 100% (if obs is less than 15)
soil1_simp	Simpson's diversity index for the soil texture type occupied by all records of each species.
geo_simp	Simpson's diversity index for the primary geological material occupied by all records of each species.
seedwt	The average total weight (in grams) of 1,000 seeds. Fern spore weight was estimated based on largest spore weight recorded in Gomez-Noguez et al. 2016
woody	Is the plant woody? yes or no.
dispersal	Dispersal type. none (seeds just drop), animal (birds or mammals eat the seeds), wind/none (tiny seeds that either fly on the wind or just drop in low-wind forest understory conditions), winged (seeds with adaptations for flying)
meanlat	the mean latitude (UTM NAD1983 zone 17N) of all presence records.
maxD	maximum range extent: the largest distance between two occurrences (km) in the study region
hull_area	range area: the total area of a convex hull enclosing all known occurrences in the study region (ha)
density	occurrence density: totpres divided by hull_area

Table S4: Species distribution accuracy measures and trait data for all species in the analysis

species	family	model	reg	obs	abs_indep	pres_indep	pres_survey	totpres	AUC_test	AUC_indep	TPR_mpa	soil1_simp	geo_simp	seedwt	woody	dispersal	meanlat	maxD	hull_area	density
Aplectrum hyemale	Orchidaceae	A	A	5	48	10	0	22	0.9944	0.6313	0.4000	0.5390635	0.4958679	0.0015	no	wind/none	4823988	332073	28824	0.076325
Aplectrum hyemale	Orchidaceae	A	B	5	48	10	0	22	0.9960	0.6313	0.4000	0.5390635	0.4958679	0.0015	no	wind/none	4823988	332073	28824	0.076325
Aplectrum hyemale	Orchidaceae	B	A	5	38	10	0	22	0.9967	0.5868	0.4000	0.5390635	0.4958679	0.0015	no	wind/none	4823988	332073	28824	0.076325
Aplectrum hyemale	Orchidaceae	B	B	5	38	10	0	22	0.9940	0.6368	0.4000	0.5390635	0.4958679	0.0015	no	wind/none	4823988	332073	28824	0.076325
Aplectrum hyemale	Orchidaceae	C	A	5	48	10	0	22	0.9973	0.6313	0.4000	0.5390635	0.4958679	0.0015	no	wind/none	4823988	332073	28824	0.076325
Aplectrum hyemale	Orchidaceae	C	B	5	48	10	0	22	0.9929	0.6563	0.4000	0.5390635	0.4958679	0.0015	no	wind/none	4823988	332073	28824	0.076325
Aplectrum hyemale	Orchidaceae	D	A	5	38	10	0	22	0.9948	0.6053	0.4000	0.5390635	0.4958679	0.0015	no	wind/none	4823988	332073	28824	0.076325
Aplectrum hyemale	Orchidaceae	D	B	5	38	10	0	22	0.9914	0.7342	0.4000	0.5390635	0.4958679	0.0015	no	wind/none	4823988	332073	28824	0.076325
Arisaema dracontium	Araceae	A	A	73	151	99	5	180	0.9743	0.6720	0.6465	0.591888	0.7285185	50.1150	no	animal	4763055	354270	30616	0.587928
Arisaema dracontium	Araceae	A	B	73	151	99	5	180	0.9723	0.6860	0.6162	0.591888	0.7285185	50.1150	no	animal	4763055	354270	30616	0.587928
Arisaema dracontium	Araceae	B	A	73	141	99	5	180	0.9857	0.5947	0.7778	0.591888	0.7285185	50.1150	no	animal	4763055	354270	30616	0.587928
Arisaema dracontium	Araceae	B	B	73	141	99	5	180	0.9850	0.6084	0.6465	0.591888	0.7285185	50.1150	no	animal	4763055	354270	30616	0.587928
Arisaema dracontium	Araceae	C	A	73	151	99	5	180	0.9872	0.7057	0.6970	0.591888	0.7285185	50.1150	no	animal	4763055	354270	30616	0.587928
Arisaema dracontium	Araceae	C	B	73	151	99	5	180	0.9862	0.7243	0.7071	0.591888	0.7285185	50.1150	no	animal	4763055	354270	30616	0.587928
Arisaema dracontium	Araceae	D	A	73	141	99	5	180	0.9898	0.6783	0.7576	0.591888	0.7285185	50.1150	no	animal	4763055	354270	30616	0.587928
Arisaema dracontium	Araceae	D	B	73	141	99	5	180	0.9862	0.7021	0.6768	0.591888	0.7285185	50.1150	no	animal	4763055	354270	30616	0.587928
Asimina triloba	Annonaceae	A	A	43	156	16	0	70	0.9823	0.7548	0.6450	0.691358	0.6570048	847.0000	yes	animal	4723402	342082	20127	0.347792
Asimina triloba	Annonaceae	A	B	43	156	16	0	70	0.9818	0.7508	0.3750	0.691358	0.6570048	847.0000	yes	animal	4723402	342082	20127	0.347792
Asimina triloba	Annonaceae	B	A	43	146	16	0	70	0.9765	0.7573	0.5	0.691358	0.6570048	847.0000	yes	animal	4723402	342082	20127	0.347792
Asimina triloba	Annonaceae	B	B	43	146	16	0	70	0.9767	0.7427	0.5	0.691358	0.6570048	847.0000	yes	animal	4723402	342082	20127	0.347792
Asimina triloba	Annonaceae	C	A	43	156	16	0	70	0.9849	0.7788	0.6875	0.691358	0.6570048	847.0000	yes	animal	4723402	342082	20127	0.347792
Asimina triloba	Annonaceae	C	B	43	156	16	0	70	0.9829	0.7941	0.6250	0.691358	0.6570048	847.0000	yes	animal	4723402	342082	20127	0.347792
Asimina triloba	Annonaceae	D	A	43	146	16	0	70	0.9822	0.8052	0.5	0.691358	0.6570048	847.0000	yes	animal	4723402	342082	20127	0.347792
Asimina triloba	Annonaceae	D	B	43	146	16	0	70	0.9776	0.7817	0.4375	0.691358	0.6570048	847.0000	yes	animal	4723402	342082	20127	0.347792
Asplenium scolopendrium var. americanum	Aspleniaceae	A	A	144	153	21	3	166	0.9827	0.9409	0.7619	0.5585308	0.6181594	0.0001	no	wind/none	4930190	214614	6342	2.617471
Asplenium scolopendrium var. americanum	Aspleniaceae	A	B	144	153	21	3	166	0.9861	0.9490	0.7619	0.5585308	0.6181594	0.0001	no	wind/none	4930190	214614	6342	2.617471
Asplenium scolopendrium var. americanum	Aspleniaceae	B	A	144	143	21	3	166	0.9849	0.9457	0.7619	0.5585308	0.6181594	0.0001	no	wind/none	4930190	214614	6342	2.617471
Asplenium scolopendrium var. americanum	Aspleniaceae	B	B	144	143	21	3	166	0.9827	0.9547	0.7619	0.5585308	0.6181594	0.0001	no	wind/none	4930190	214614	6342	2.617471
Asplenium scolopendrium var. americanum	Aspleniaceae	C	A	144	153	21	3	166	0.9854	0.9807	0.7619	0.5585308	0.6181594	0.0001	no	wind/none	4930190	214614	6342	2.617471
Asplenium scolopendrium var. americanum	Aspleniaceae	C	B	144	153	21	3	166	0.9875	0.9642	0.7619	0.5585308	0.6181594	0.0001	no	wind/none	4930190	214614	6342	2.617471
Asplenium scolopendrium var. americanum	Aspleniaceae	D	A	144	143	21	3	166	0.9848	0.9800	0.7619	0.5585308	0.6181594	0.0001	no	wind/none	4930190	214614	6342	2.617471

species	family	model	reg	obs	abs_indep	pres_indep	pres_survey	totpres	AUC_test	AUC_indep	TPR_mpa	soil1_simp	geo_simp	seedwt	woody	dispersal	meanlat	maxD	hull_area	density
Asplenium scolopendrium var. americanum	Aspleniaceae	D	B	144	143	21	3	166	0.9842	0.9570	0.7143	0.5585308	0.6181594	0.0001	no	wind/none	4930190	214614	6342	2.617471
Castanea dentata	Fagaceae	A	A	153	134	275	7	460	0.9744	0.8129	0.8909	0.6023963	0.5374951	3467.3	yes	animal	4744171	340349	20210	2.276101
Castanea dentata	Fagaceae	A	B	153	134	275	7	460	0.9739	0.8374	0.7673	0.6023963	0.5374951	3467.3	yes	animal	4744171	340349	20210	2.276101
Castanea dentata	Fagaceae	B	A	153	124	275	7	460	0.9778	0.8127	0.9055	0.6023963	0.5374951	3467.3	yes	animal	4744171	340349	20210	2.276101
Castanea dentata	Fagaceae	B	B	153	124	275	7	460	0.9746	0.8287	0.8073	0.6023963	0.5374951	3467.3	yes	animal	4744171	340349	20210	2.276101
Castanea dentata	Fagaceae	C	A	153	134	275	7	460	0.9828	0.8426	0.8918	0.6023963	0.5374951	3467.3	yes	animal	4744171	340349	20210	2.276101
Castanea dentata	Fagaceae	C	B	153	134	275	7	460	0.9849	0.8537	0.8582	0.6023963	0.5374951	3467.3	yes	animal	4744171	340349	20210	2.276101
Castanea dentata	Fagaceae	D	A	153	124	275	7	460	0.9827	0.8399	0.9018	0.6023963	0.5374951	3467.3	yes	animal	4744171	340349	20210	2.276101
Castanea dentata	Fagaceae	D	B	153	124	275	7	460	0.9832	0.8376	0.8036	0.6023963	0.5374951	3467.3	yes	animal	4744171	340349	20210	2.276101
Celtis tenuifolia	Ulmaceae	A	A	73	141	17	0	118	0.9929	0.9591	0.5294	0.4772238	0.7599828	93.6544	yes	animal	4754197	532297	36812	0.320548
Celtis tenuifolia	Ulmaceae	A	B	73	141	17	0	118	0.9932	0.9675	0.7059	0.4772238	0.7599828	93.6544	yes	animal	4754197	532297	36812	0.320548
Celtis tenuifolia	Ulmaceae	B	A	73	131	17	0	118	0.9936	0.9560	0.7059	0.4772238	0.7599828	93.6544	yes	animal	4754197	532297	36812	0.320548
Celtis tenuifolia	Ulmaceae	B	B	73	131	17	0	118	0.9936	0.9672	0.6471	0.4772238	0.7599828	93.6544	yes	animal	4754197	532297	36812	0.320548
Celtis tenuifolia	Ulmaceae	C	A	73	141	17	0	118	0.9931	0.9395	0.5882	0.4772238	0.7599828	93.6544	yes	animal	4754197	532297	36812	0.320548
Celtis tenuifolia	Ulmaceae	C	B	73	141	17	0	118	0.9963	0.9604	0.5294	0.4772238	0.7599828	93.6544	yes	animal	4754197	532297	36812	0.320548
Celtis tenuifolia	Ulmaceae	D	A	73	131	17	0	118	0.9935	0.9349	0.5294	0.4772238	0.7599828	93.6544	yes	animal	4754197	532297	36812	0.320548
Celtis tenuifolia	Ulmaceae	D	B	73	131	17	0	118	0.9935	0.9627	0.5294	0.4772238	0.7599828	93.6544	yes	animal	4754197	532297	36812	0.320548
Cornus florida	Cornaceae	A	A	295	147	89	8	465	0.9753	0.7602	0.6629	0.6337963	0.759624	102.0000	yes	animal	4761902	348366	32577	1.427387
Cornus florida	Cornaceae	A	B	295	147	89	8	465	0.9726	0.7859	0.6854	0.6337963	0.759624	102.0000	yes	animal	4761902	348366	32577	1.427387
Cornus florida	Cornaceae	B	A	295	137	89	8	465	0.9745	0.7281	0.6629	0.6337963	0.759624	102.0000	yes	animal	4761902	348366	32577	1.427387
Cornus florida	Cornaceae	B	B	295	137	89	8	465	0.9744	0.7750	0.7191	0.6337963	0.759624	102.0000	yes	animal	4761902	348366	32577	1.427387
Cornus florida	Cornaceae	C	A	295	147	89	8	465	0.9807	0.7289	0.3933	0.6337963	0.759624	102.0000	yes	animal	4761902	348366	32577	1.427387
Cornus florida	Cornaceae	C	B	295	147	89	8	465	0.9803	0.7787	0.5506	0.6337963	0.759624	102.0000	yes	animal	4761902	348366	32577	1.427387
Cornus florida	Cornaceae	D	A	295	137	89	8	465	0.9794	0.6937	0.4045	0.6337963	0.759624	102.0000	yes	animal	4761902	348366	32577	1.427387
Cornus florida	Cornaceae	D	B	295	137	89	8	465	0.9800	0.7312	0.4719	0.6337963	0.759624	102.0000	yes	animal	4761902	348366	32577	1.427387
Cypripedium arietinum	Orchidaceae	A	A	77	41	27	0	109	0.9227	0.8600	0.9259	0.749021	0.7325983	0.0019	no	wind/none	4964400	621692	91478	0.119154
Cypripedium arietinum	Orchidaceae	A	B	77	41	27	0	109	0.9418	0.8229	0.8519	0.749021	0.7325983	0.0019	no	wind/none	4964400	621692	91478	0.119154
Cypripedium arietinum	Orchidaceae	B	A	65	31	27	0	109	0.9411	0.8411	0.8148	0.749021	0.7325983	0.0019	no	wind/none	4964400	621692	91478	0.119154
Cypripedium arietinum	Orchidaceae	B	B	65	31	27	0	109	0.9646	0.8608	0.8148	0.749021	0.7325983	0.0019	no	wind/none	4964400	621692	91478	0.119154
Cypripedium arietinum	Orchidaceae	C	A	77	41	27	0	109	0.9565	0.8157	0.8519	0.749021	0.7325983	0.0019	no	wind/none	4964400	621692	91478	0.119154
Cypripedium arietinum	Orchidaceae	C	B	77	41	27	0	109	0.9442	0.8076	0.8889	0.749021	0.7325983	0.0019	no	wind/none	4964400	621692	91478	0.119154
Cypripedium arietinum	Orchidaceae	D	A	65	31	27	0	109	0.9669	0.8686	0.7407	0.749021	0.7325983	0.0019	no	wind/none	4964400	621692	91478	0.119154
Cypripedium arietinum	Orchidaceae	D	B	65	31	27	0	109	0.9661	0.8692	0.8148	0.749021	0.7325983	0.0019	no	wind/none	4964400	621692	91478	0.119154
Enemion biternatum	Ranunculaceae	A	A	14	57	26	0	49	0.9868	0.8576	0.8077	0.37875	0.6397334	2.7672	no	none	4755022	69626	1141	4.294479
Enemion biternatum	Ranunculaceae	A	B	14	57	26	0	49	0.9931	0.8617	0.7308	0.37875	0.6397334	2.7672	no	none	4755022	69626	1141	4.294479
Enemion biternatum	Ranunculaceae	B	A	14	47	26	0	49	0.9902	0.8208	0.7308	0.37875	0.6397334	2.7672	no	none	4755022	69626	1141	4.294479
Enemion biternatum	Ranunculaceae	B	B	14	47	26	0	49	0.9822	0.8028	0.6923	0.37875	0.6397334	2.7672	no	none	4755022	69626	1141	4.294479

species	family	model	reg	obs	abs_indep	pres_indep	pres_survey	totpres	AUC_test	AUC_indep	TPR_mpa	soil1_simp	geo_simp	seedwt	woody	dispersal	meanlat	maxD	hull_area	density
Enemion biternatum	Ranunculaceae	C	A	14	57	26	0	49	0.9844	0.8623	0.9231	0.37875	0.6397334	2.7672	no	none	4755022	69626	1141	4.294479
Enemion biternatum	Ranunculaceae	C	B	14	57	26	0	49	0.9928	0.8873	0.8077	0.37875	0.6397334	2.7672	no	none	4755022	69626	1141	4.294479
Enemion biternatum	Ranunculaceae	D	A	14	47	26	0	49	0.9893	0.8347	0.7308	0.37875	0.6397334	2.7672	no	none	4755022	69626	1141	4.294479
Enemion biternatum	Ranunculaceae	D	B	14	47	26	0	49	0.9838	0.8224	0.6923	0.37875	0.6397334	2.7672	no	none	4755022	69626	1141	4.294479
Erigenia bulbosa	Apiaceae	A	A	16	23	12	0	30	0.91	0.4529	0.8333	0.6397146	0.7488889	2.2570	no	none	4756502	322944	13945	0.215131
Erigenia bulbosa	Apiaceae	A	B	16	23	12	0	30	0.919	0.4638	0.5000	0.6397146	0.7488889	2.2570	no	none	4756502	322944	13945	0.215131
Erigenia bulbosa	Apiaceae	B	A	16	23	12	0	30	0.9855	0.4529	0.4167	0.6397146	0.7488889	2.2570	no	none	4756502	322944	13945	0.215131
Erigenia bulbosa	Apiaceae	B	B	16	23	12	0	30	0.973	0.4457	0.2500	0.6397146	0.7488889	2.2570	no	none	4756502	322944	13945	0.215131
Erigenia bulbosa	Apiaceae	C	A	16	23	12	0	30	0.8940	0.5217	0.5000	0.6397146	0.7488889	2.2570	no	none	4756502	322944	13945	0.215131
Erigenia bulbosa	Apiaceae	C	B	16	23	12	0	30	0.8763	0.5399	0.5833	0.6397146	0.7488889	2.2570	no	none	4756502	322944	13945	0.215131
Erigenia bulbosa	Apiaceae	D	A	16	23	12	0	30	0.9586	0.3877	0.5000	0.6397146	0.7488889	2.2570	no	none	4756502	322944	13945	0.215131
Erigenia bulbosa	Apiaceae	D	B	16	23	12	0	30	0.9774	0.4384	0.3333	0.6397146	0.7488889	2.2570	no	none	4756502	322944	13945	0.215131
Eurybia divaricata	Asteraceae	A	A	42	42	11	0	74	0.9944	0.8571	0.2727	0.2363831	0.535608	0.6443	no	wind/none	4769268	65678	1734	4.267589
Eurybia divaricata	Asteraceae	A	B	42	42	11	0	74	0.9913	0.8745	0.2727	0.2363831	0.535608	0.6443	no	wind/none	4769268	65678	1734	4.267589
Eurybia divaricata	Asteraceae	B	A	42	42	11	0	74	0.9938	0.8810	0.2727	0.2363831	0.535608	0.6443	no	wind/none	4769268	65678	1734	4.267589
Eurybia divaricata	Asteraceae	B	B	42	42	11	0	74	0.9941	0.8506	0.2727	0.2363831	0.535608	0.6443	no	wind/none	4769268	65678	1734	4.267589
Eurybia divaricata	Asteraceae	C	A	42	42	11	0	74	0.9966	0.8874	0.1818	0.2363831	0.535608	0.6443	no	wind/none	4769268	65678	1734	4.267589
Eurybia divaricata	Asteraceae	C	B	42	42	11	0	74	0.9951	0.9004	0.2727	0.2363831	0.535608	0.6443	no	wind/none	4769268	65678	1734	4.267589
Eurybia divaricata	Asteraceae	D	A	42	42	11	0	74	0.9964	0.8636	0.4545	0.2363831	0.535608	0.6443	no	wind/none	4769268	65678	1734	4.267589
Eurybia divaricata	Asteraceae	D	B	42	42	11	0	74	0.9949	0.9091	0.4545	0.2363831	0.535608	0.6443	no	wind/none	4769268	65678	1734	4.267589
Frasera caroliniensis	Gentianaceae	A	A	30	155	19	0	50	0.9947	0.8995	0.8421	0.4789671	0.764	9.4498	no	none	4782447	101791	2995	1.669449
Frasera caroliniensis	Gentianaceae	A	B	30	155	19	0	50	0.9953	0.9182	0.7368	0.4789671	0.764	9.4498	no	none	4782447	101791	2995	1.669449
Frasera caroliniensis	Gentianaceae	B	A	30	145	19	0	50	0.9953	0.8715	0.8421	0.4789671	0.764	9.4498	no	none	4782447	101791	2995	1.669449
Frasera caroliniensis	Gentianaceae	B	B	30	145	19	0	50	0.9963	0.8762	0.7895	0.4789671	0.764	9.4498	no	none	4782447	101791	2995	1.669449
Frasera caroliniensis	Gentianaceae	C	A	30	155	19	0	50	0.9972	0.8927	0.7368	0.4789671	0.764	9.4498	no	none	4782447	101791	2995	1.669449
Frasera caroliniensis	Gentianaceae	C	B	30	155	19	0	50	0.9972	0.8815	0.7895	0.4789671	0.764	9.4498	no	none	4782447	101791	2995	1.669449
Frasera caroliniensis	Gentianaceae	D	A	30	145	19	0	50	0.9976	0.8817	0.8947	0.4789671	0.764	9.4498	no	none	4782447	101791	2995	1.669449
Frasera caroliniensis	Gentianaceae	D	B	30	145	19	0	50	0.9978	0.8584	0.5789	0.4789671	0.764	9.4498	no	none	4782447	101791	2995	1.669449
Fraxinus quadrangulata	Oleaceae	A	A	46	131	65	1	194	0.9916	0.8652	0.7385	0.7257651	0.8365926	70.1800	yes	winged	4663494	322035	19607	0.989443
Fraxinus quadrangulata	Oleaceae	A	B	46	131	65	1	194	0.9951	0.8979	0.6769	0.7257651	0.8365926	70.1800	yes	winged	4663494	322035	19607	0.989443
Fraxinus quadrangulata	Oleaceae	B	A	46	121	65	1	194	0.994	0.8432	0.6923	0.7257651	0.8365926	70.1800	yes	winged	4663494	322035	19607	0.989443
Fraxinus quadrangulata	Oleaceae	B	B	46	121	65	1	194	0.9927	0.8479	0.6154	0.7257651	0.8365926	70.1800	yes	winged	4663494	322035	19607	0.989443
Fraxinus quadrangulata	Oleaceae	C	A	46	131	65	1	194	0.9946	0.8162	0.6615	0.7257651	0.8365926	70.1800	yes	winged	4663494	322035	19607	0.989443
Fraxinus quadrangulata	Oleaceae	C	B	46	131	65	1	194	0.9958	0.8550	0.6154	0.7257651	0.8365926	70.1800	yes	winged	4663494	322035	19607	0.989443
Fraxinus quadrangulata	Oleaceae	D	A	46	121	65	1	194	0.9933	0.8257	0.7077	0.7257651	0.8365926	70.1800	yes	winged	4663494	322035	19607	0.989443
Fraxinus quadrangulata	Oleaceae	D	B	46	121	65	1	194	0.9943	0.8563	0.5692	0.7257651	0.8365926	70.1800	yes	winged	4663494	322035	19607	0.989443
Gymnocladus dioicus	Fabaceae	A	A	23	131	21	0	200	0.9848	0.6419	0.2381	0.7204177	0.8317725	1843.0	yes	none	4658654	345225	23485	0.851607
Gymnocladus dioicus	Fabaceae	A	B	23	131	21	0	200	0.9818	0.6361	0.0476	0.7204177	0.8317725	1843.0	yes	none	4658654	345225	23485	0.851607
Gymnocladus dioicus	Fabaceae	B	A	23	131	21	0	200	0.9807	0.6529	0.1905	0.7204177	0.8317725	1843.0	yes	none	4658654	345225	23485	0.851607

species	family	model	reg	obs	abs_indep	pres_indep	pres_survey	totpres	AUC_test	AUC_indep	TPR_mpa	soil1_simp	geo_simp	seedwt	woody	dispersal	meanlat	maxD	hull_area	density
Gymnocladus dioicus	Fabaceae	B	B	23	131	21	0	200	0.9806	0.6209	0.0952	0.7204177	0.8317725	1843.0	yes	none	4658654	345225	23485	0.851607
Gymnocladus dioicus	Fabaceae	C	A	23	131	21	0	200	0.9835	0.6401	0.1429	0.7204177	0.8317725	1843.0	yes	none	4658654	345225	23485	0.851607
Gymnocladus dioicus	Fabaceae	C	B	23	131	21	0	200	0.9885	0.6140	0.0952	0.7204177	0.8317725	1843.0	yes	none	4658654	345225	23485	0.851607
Gymnocladus dioicus	Fabaceae	D	A	23	131	21	0	200	0.9835	0.6194	0.1429	0.7204177	0.8317725	1843.0	yes	none	4658654	345225	23485	0.851607
Gymnocladus dioicus	Fabaceae	D	B	23	131	21	0	200	0.9829	0.6118	0.0000	0.7204177	0.8317725	1843.0	yes	none	4658654	345225	23485	0.851607
Heuchera americana	Saxifragaceae	A	A	19	156	12	0	34	0.9848	0.9097	0.5000	0.5692042	0.6384083	0.0252	no	wind/none	4638165	64597	499	6.813627
Heuchera americana	Saxifragaceae	A	B	19	156	12	0	34	0.9969	0.9225	0.5000	0.5692042	0.6384083	0.0252	no	wind/none	4638165	64597	499	6.813627
Heuchera americana	Saxifragaceae	B	A	19	146	12	0	34	0.9729	0.9304	0.7500	0.5692042	0.6384083	0.0252	no	wind/none	4638165	64597	499	6.813627
Heuchera americana	Saxifragaceae	B	B	19	146	12	0	34	0.9743	0.9492	0.5000	0.5692042	0.6384083	0.0252	no	wind/none	4638165	64597	499	6.813627
Heuchera americana	Saxifragaceae	C	A	19	156	12	0	34	0.9909	0.9236	0.4167	0.5692042	0.6384083	0.0252	no	wind/none	4638165	64597	499	6.813627
Heuchera americana	Saxifragaceae	C	B	19	156	12	0	34	0.9914	0.9343	0.5000	0.5692042	0.6384083	0.0252	no	wind/none	4638165	64597	499	6.813627
Heuchera americana	Saxifragaceae	D	A	19	146	12	0	34	0.9809	0.9418	0.6667	0.5692042	0.6384083	0.0252	no	wind/none	4638165	64597	499	6.813627
Heuchera americana	Saxifragaceae	D	B	19	146	12	0	34	0.9735	0.9338	0.4444	0.5692042	0.6384083	0.0252	no	wind/none	4638165	64597	499	6.813627
Hydrastis canadensis	Ranunculaceae	A	A	44	156	35	0	90	0.9682	0.8057	0.6571	0.5668476	0.7780247	10.9036	no	animal	4732533	328255	26736	0.336625
Hydrastis canadensis	Ranunculaceae	A	B	44	156	35	0	90	0.9533	0.8258	0.6571	0.5668476	0.7780247	10.9036	no	animal	4732533	328255	26736	0.336625
Hydrastis canadensis	Ranunculaceae	B	A	44	146	35	0	90	0.9806	0.7955	0.5429	0.5668476	0.7780247	10.9036	no	animal	4732533	328255	26736	0.336625
Hydrastis canadensis	Ranunculaceae	B	B	44	146	35	0	90	0.9762	0.8485	0.6000	0.5668476	0.7780247	10.9036	no	animal	4732533	328255	26736	0.336625
Hydrastis canadensis	Ranunculaceae	C	A	44	156	35	0	90	0.9861	0.8332	0.6000	0.5668476	0.7780247	10.9036	no	animal	4732533	328255	26736	0.336625
Hydrastis canadensis	Ranunculaceae	C	B	44	156	35	0	90	0.9865	0.8535	0.5429	0.5668476	0.7780247	10.9036	no	animal	4732533	328255	26736	0.336625
Hydrastis canadensis	Ranunculaceae	D	A	44	146	35	0	90	0.9850	0.8153	0.4571	0.5668476	0.7780247	10.9036	no	animal	4732533	328255	26736	0.336625
Hydrastis canadensis	Ranunculaceae	D	B	44	146	35	0	90	0.9845	0.8188	0.5429	0.5668476	0.7780247	10.9036	no	animal	4732533	328255	26736	0.336625
Juglans cinerea	Juglandaceae	A	A	1888	138	522	3	2723	0.8604	0.4663	0.4023	0.4979165	0.7769754	14026.0	yes	animal	4955907	790142	141847	1.919674
Juglans cinerea	Juglandaceae	A	B	1888	138	522	3	2723	0.8662	0.4553	0.3257	0.4979165	0.7769754	14026.0	yes	animal	4955907	790142	141847	1.919674
Juglans cinerea	Juglandaceae	B	A	1594	129	513	3	2723	0.8806	0.4453	0.4366	0.4979165	0.7769754	14026.0	yes	animal	4955907	790142	141847	1.919674
Juglans cinerea	Juglandaceae	B	B	1594	129	513	3	2723	0.8865	0.4305	0.3665	0.4979165	0.7769754	14026.0	yes	animal	4955907	790142	141847	1.919674
Juglans cinerea	Juglandaceae	C	A	1888	138	522	3	2723	0.8743	0.4824	0.4636	0.4979165	0.7769754	14026.0	yes	animal	4955907	790142	141847	1.919674
Juglans cinerea	Juglandaceae	C	B	1888	138	522	3	2723	0.8736	0.4458	0.4061	0.4979165	0.7769754	14026.0	yes	animal	4955907	790142	141847	1.919674
Juglans cinerea	Juglandaceae	D	A	1594	129	513	3	2723	0.8810	0.4670	0.4854	0.4979165	0.7769754	14026.0	yes	animal	4955907	790142	141847	1.919674
Juglans cinerea	Juglandaceae	D	B	1594	129	513	3	2723	0.8907	0.4417	0.3918	0.4979165	0.7769754	14026.0	yes	animal	4955907	790142	141847	1.919674
Liparis liliifolia	Orchidaceae	A	A	16	133	53	0	74	0.8891	0.9628	0.9434	0.4181885	0.5865595	0.0040	no	wind/none	4702675	594586	32507	0.227643
Liparis liliifolia	Orchidaceae	A	B	16	133	53	0	74	0.9171	0.9625	0.9434	0.4181885	0.5865595	0.0040	no	wind/none	4702675	594586	32507	0.227643
Liparis liliifolia	Orchidaceae	B	A	16	123	53	0	74	0.9705	0.9646	0.9434	0.4181885	0.5865595	0.0040	no	wind/none	4702675	594586	32507	0.227643
Liparis liliifolia	Orchidaceae	B	B	16	123	53	0	74	0.9692	0.9612	0.9245	0.4181885	0.5865595	0.0040	no	wind/none	4702675	594586	32507	0.227643
Liparis liliifolia	Orchidaceae	C	A	16	133	53	0	74	0.9566	0.9699	0.9623	0.4181885	0.5865595	0.0040	no	wind/none	4702675	594586	32507	0.227643
Liparis liliifolia	Orchidaceae	C	B	16	133	53	0	74	0.9479	0.9664	0.9434	0.4181885	0.5865595	0.0040	no	wind/none	4702675	594586	32507	0.227643
Liparis liliifolia	Orchidaceae	D	A	16	123	53	0	74	0.9850	0.9712	0.9623	0.4181885	0.5865595	0.0040	no	wind/none	4702675	594586	32507	0.227643
Liparis liliifolia	Orchidaceae	D	B	16	123	53	0	74	0.9455	0.9693	0.8868	0.4181885	0.5865595	0.0040	no	wind/none	4702675	594586	32507	0.227643
Lithospermum latifolium	Boraginaceae	A	A	11	136	23	5	37	0.9630	0.6563	0.7391	0.5518367	0.7406866	21.7810	no	none	4779621	189202	12497	0.296071
Lithospermum latifolium	Boraginaceae	A	B	11	136	23	5	37	0.9306	0.6643	0.7391	0.5518367	0.7406866	21.7810	no	none	4779621	189202	12497	0.296071
Lithospermum latifolium	Boraginaceae	B	A	11	126	23	5	37	0.9964	0.6370	0.4783	0.5518367	0.7406866	21.7810	no	none	4779621	189202	12497	0.296071
Lithospermum latifolium	Boraginaceae	B	B	11	126	23	5	37	0.9792	0.6070	0.2609	0.5518367	0.7406866	21.7810	no	none	4779621	189202	12497	0.296071

species	family	model	reg	obs	abs_indep	pres_indep	pres_survey	totpres	AUC_test	AUC_indep	TPR_mpa	soil1_simp	geo_simp	seedwt	woody	dispersal	meanlat	maxD	hull_area	density
Lithospermum latifolium	Boraginaceae	C	A	11	136	23	5	37	0.9311	0.7008	0.6960	0.5518367	0.7406866	21.7810	no	none	4779621	189202	12497	0.296071
Lithospermum latifolium	Boraginaceae	C	B	11	136	23	5	37	0.9234	0.7017	0.6957	0.5518367	0.7406866	21.7810	no	none	4779621	189202	12497	0.296071
Lithospermum latifolium	Boraginaceae	D	A	11	126	23	5	37	0.9754	0.6563	0.6087	0.5518367	0.7406866	21.7810	no	none	4779621	189202	12497	0.296071
Lithospermum latifolium	Boraginaceae	D	B	11	126	23	5	37	0.9721	0.5769	0.2609	0.5518367	0.7406866	21.7810	no	none	4779621	189202	12497	0.296071
Magnolia acuminata	Magnoliaceae	A	A	58	155	45	0	111	0.9873	0.8449	0.4222	0.6343764	0.5080756	88.5100	yes	animal	4743870	251223	30341	0.365842
Magnolia acuminata	Magnoliaceae	A	B	58	155	45	0	111	0.9751	0.8687	0.4889	0.6343764	0.5080756	88.5100	yes	animal	4743870	251223	30341	0.365842
Magnolia acuminata	Magnoliaceae	B	A	58	145	45	0	111	0.9854	0.8061	0.6444	0.6343764	0.5080756	88.5100	yes	animal	4743870	251223	30341	0.365842
Magnolia acuminata	Magnoliaceae	B	B	58	145	45	0	111	0.9849	0.8303	0.2667	0.6343764	0.5080756	88.5100	yes	animal	4743870	251223	30341	0.365842
Magnolia acuminata	Magnoliaceae	C	A	58	155	45	0	111	0.9885	0.8684	0.5333	0.6343764	0.5080756	88.5100	yes	animal	4743870	251223	30341	0.365842
Magnolia acuminata	Magnoliaceae	C	B	58	155	45	0	111	0.9726	0.8839	0.6000	0.6343764	0.5080756	88.5100	yes	animal	4743870	251223	30341	0.365842
Magnolia acuminata	Magnoliaceae	D	A	58	145	45	0	111	0.9818	0.8225	0.3111	0.6343764	0.5080756	88.5100	yes	animal	4743870	251223	30341	0.365842
Magnolia acuminata	Magnoliaceae	D	B	58	145	45	0	111	0.9892	0.8736	0.4222	0.6343764	0.5080756	88.5100	yes	animal	4743870	251223	30341	0.365842
Mertensia virginica	Boraginaceae	A	A	6	51	28	2	36	0.9307	0.8284	0.3571	0.4152249	0.7134694	2.9223	no	none	4744859	257262	13512	0.26643
Mertensia virginica	Boraginaceae	A	B	6	51	28	2	36	0.9217	0.7983	0.2500	0.4152249	0.7134694	2.9223	no	none	4744859	257262	13512	0.26643
Mertensia virginica	Boraginaceae	B	A	6	41	28	2	36	0.9420	0.7143	0.3571	0.4152249	0.7134694	2.9223	no	none	4744859	257262	13512	0.26643
Mertensia virginica	Boraginaceae	B	B	6	41	28	2	36	0.9920	0.8066	0.2857	0.4152249	0.7134694	2.9223	no	none	4744859	257262	13512	0.26643
Mertensia virginica	Boraginaceae	C	A	6	51	28	2	36	0.9744	0.8578	0.3571	0.4152249	0.7134694	2.9223	no	none	4744859	257262	13512	0.26643
Mertensia virginica	Boraginaceae	C	B	6	51	28	2	36	0.9473	0.8326	0.2500	0.4152249	0.7134694	2.9223	no	none	4744859	257262	13512	0.26643
Mertensia virginica	Boraginaceae	D	A	6	41	28	2	36	0.9471	0.7065	0.3214	0.4152249	0.7134694	2.9223	no	none	4744859	257262	13512	0.26643
Mertensia virginica	Boraginaceae	D	B	6	41	28	2	36	0.9896	0.7274	0.2857	0.4152249	0.7134694	2.9223	no	none	4744859	257262	13512	0.26643
Nyssa sylvatica	Nyssaceae	A	A	21	140	29	2	68	0.9752	0.8017	0.3103	0.652562	0.6422222	140.0000	yes	animal	4731319	345874	17862	0.380696
Nyssa sylvatica	Nyssaceae	A	B	21	140	29	2	68	0.9657	0.7909	0.3448	0.652562	0.6422222	140.0000	yes	animal	4731319	345874	17862	0.380696
Nyssa sylvatica	Nyssaceae	B	A	21	140	29	2	68	0.9758	0.8369	0.6207	0.652562	0.6422222	140.0000	yes	animal	4731319	345874	17862	0.380696
Nyssa sylvatica	Nyssaceae	B	B	21	140	29	2	68	0.9665	0.8091	0.4483	0.652562	0.6422222	140.0000	yes	animal	4731319	345874	17862	0.380696
Nyssa sylvatica	Nyssaceae	C	A	21	140	29	2	68	0.9873	0.8441	0.3793	0.652562	0.6422222	140.0000	yes	animal	4731319	345874	17862	0.380696
Nyssa sylvatica	Nyssaceae	C	B	21	140	29	2	68	0.9841	0.8502	0.2414	0.652562	0.6422222	140.0000	yes	animal	4731319	345874	17862	0.380696
Nyssa sylvatica	Nyssaceae	D	A	21	140	29	2	68	0.9847	0.8345	0.3103	0.652562	0.6422222	140.0000	yes	animal	4731319	345874	17862	0.380696
Nyssa sylvatica	Nyssaceae	D	B	21	140	29	2	68	0.9868	0.8438	0.3103	0.652562	0.6422222	140.0000	yes	animal	4731319	345874	17862	0.380696
Panax quinquefolius	Araliaceae	A	A	490	111	167	2	751	0.8986	0.8187	0.9222	0.3724247	0.4268125	27.7540	no	animal	4941323	700882	113977	0.658905
Panax quinquefolius	Araliaceae	A	B	490	111	167	2	751	0.9078	0.7846	0.8862	0.3724247	0.4268125	27.7540	no	animal	4941323	700882	113977	0.658905
Panax quinquefolius	Araliaceae	B	A	214	111	57	2	751	0.9360	0.7044	0.9298	0.3724247	0.4268125	27.7540	no	animal	4941323	700882	113977	0.658905
Panax quinquefolius	Araliaceae	B	B	214	111	57	2	751	0.9358	0.7658	0.8719	0.3724247	0.4268125	27.7540	no	animal	4941323	700882	113977	0.658905
Panax quinquefolius	Araliaceae	C	A	490	111	167	2	751	0.9311	0.8480	0.8982	0.3724247	0.4268125	27.7540	no	animal	4941323	700882	113977	0.658905
Panax quinquefolius	Araliaceae	C	B	490	111	167	2	751	0.9332	0.8047	0.8802	0.3724247	0.4268125	27.7540	no	animal	4941323	700882	113977	0.658905
Panax quinquefolius	Araliaceae	D	A	214	111	57	2	751	0.9504	0.7697	0.8772	0.3724247	0.4268125	27.7540	no	animal	4941323	700882	113977	0.658905
Panax quinquefolius	Araliaceae	D	B	214	111	57	2	751	0.9544	0.8095	0.8596	0.3724247	0.4268125	27.7540	no	animal	4941323	700882	113977	0.658905
Phegopteris hexagonoptera	Thelypteridaceae	A	A	38	132	10	1	53	0.9257	0.4598	0.6000	0.532872	0.7931648	0.0001	no	wind/none	4790511	635513	42453	0.124844
Phegopteris hexagonoptera	Thelypteridaceae	A	B	38	132	10	1	53	0.9276	0.4742	0.4000	0.532872	0.7931648	0.0001	no	wind/none	4790511	635513	42453	0.124844
Phegopteris hexagonoptera	Thelypteridaceae	B	A	38	122	10	1	53	0.9659	0.3984	0.4000	0.532872	0.7931648	0.0001	no	wind/none	4790511	635513	42453	0.124844

species	family	model	reg	obs	abs_indep	pres_indep	pres_survey	totpres	AUC_test	AUC_indep	TPR_mpa	soil1_simp	geo_simp	seedwt	woody	dispersal	meanlat	maxD	hull_area	density
Phegopteris hexagonoptera	Thelypteridaceae	B	B	38	122	10	1	53	0.9415	0.3566	0.3000	0.532872	0.7931648	0.0001	no	wind/none	4790511	635513	42453	0.124844
Phegopteris hexagonoptera	Thelypteridaceae	C	A	38	132	10	1	53	0.9692	0.6318	0.6000	0.532872	0.7931648	0.0001	no	wind/none	4790511	635513	42453	0.124844
Phegopteris hexagonoptera	Thelypteridaceae	C	B	38	132	10	1	53	0.9768	0.5977	0.6000	0.532872	0.7931648	0.0001	no	wind/none	4790511	635513	42453	0.124844
Phegopteris hexagonoptera	Thelypteridaceae	D	A	38	122	10	1	53	0.9972	0.4402	0.5000	0.532872	0.7931648	0.0001	no	wind/none	4790511	635513	42453	0.124844
Phegopteris hexagonoptera	Thelypteridaceae	D	B	38	122	10	1	53	0.9796	0.4410	0.1000	0.532872	0.7931648	0.0001	no	wind/none	4790511	635513	42453	0.124844

Table S5: Model selection results for the glmm with independent AUC (AUC_indep) as the response variable, showing the ranking of all models as determined by the 'dredge' function. Predictor variables are listed in the first 8 columns, with "+" indicating that the predictor was included in the model. See table S3 for explanation of predictor variable codes. df = degrees of freedom, logLik = log-likelihood.

woody	dispersal	soil1_simp	density	maxD	log (seedwt)	log $(\text{seedwt})^2$	meanlat	df	logLik	AICc	delta
+	+	+	+		+	+		12	182.7247	-339.706	0
+	+		+		+	+		11	181.1093	-338.752	0.954314
+	+	+	+	+	+	+		13	182.8245	-337.604	2.102335
+	+		+	+	+	+		12	181.2119	-336.681	3.025635
+	+		+		+	+	+	12	181.1374	-336.532	3.174526
+	+	+	+	+	+	+	+	14	182.8258	-335.279	4.42765
+	+		+	+	+	+	+	13	181.3201	-334.595	5.11106
+		+	+		+	+		9	172.9745	-326.96	12.74636
+		+	+	+	+	+		10	173.6567	-326.098	13.60848
+		+	+		+	+	+	10	173.3351	-325.455	14.25165
+			+	+	+	+		9	171.6616	-324.334	15.37213
+		+	+	+	+	+	+	11	173.7183	-323.97	15.7364
+			+	+	+	+	+	10	171.6671	-322.119	17.58764
+			+		+	+		8	169.1129	-321.439	18.26736
+			+		+	+	+	9	169.7169	-320.445	19.26144
+	+	+		+	+	+	+	13	171.4649	-314.885	24.82138
+	+	+		+	+	+		12	169.842	-313.941	25.76528
+	+	+	+	+		+		12	167.9535	-310.164	29.54241
+	+	+	+	+		+	+	13	168.8409	-309.637	30.06943
+	+	+		+		+	+	12	166.9895	-308.236	31.47044
+	+	+		+		+		11	164.628	-305.789	33.91699
	+		+		+	+	+	11	163.4226	-303.378	36.32789
	+		+	+	+	+	+	12	163.4859	-301.229	38.47748
	+	+	+		+	+	+	12	163.4427	-301.142	38.56399
	+	+	+	+	+	+	+	13	163.5829	-299.121	40.58552
+	+		+	+		+		11	161.2864	-299.106	40.60024
	+		+	+	+	+		11	160.6943	-297.922	41.78443
	+		+		+	+		10	159.4448	-297.674	42.03222

woody	dispersal	soil1_simp	density	maxD	log (seedwt)	log $(seedwt)^2$	meanlat	df	logLik	AICc	delta
+	+	+	+			+		11	160.4052	-297.344	42.36265
+	+		+	+		+	+	12	161.3338	-296.925	42.7818
	+	+	+	+	+	+		12	161.1382	-296.533	43.17291
+		+		+	+	+		9	157.5166	-296.044	43.66212
	+	+	+	+		+		11	159.7318	-295.997	43.70947
+		+		+	+	+	+	10	158.3236	-295.432	44.27463
+	+	+	+			+	+	12	160.5852	-295.427	44.2789
	+	+	+		+	+		11	159.445	-295.423	44.28309
+	+		+			+		10	158.0498	-294.884	44.82216
	+	+	+	+		+	+	12	160.3012	-294.859	44.84693
	+		+	+		+		10	157.69	-294.164	45.5419
+	+	+	+	+	+		+	13	160.7609	-293.477	46.22943
+	+	+		+	+			11	158.3719	-293.277	46.42918
+	+		+			+	+	11	158.2721	-293.077	46.62886
	+		+	+		+	+	11	158.2377	-293.009	46.69759
+	+	+	+	+	+			12	159.116	-292.489	47.21732
+	+	+		+	+		+	12	158.9131	-292.083	47.62305
+	+			+	+	+	+	12	158.8692	-291.995	47.71102
+	+			+	+	+		11	157.7122	-291.958	47.74868
	+	+		+		+		10	156.2892	-291.363	48.34345
	+		+			+	+	10	156.0916	-290.968	48.73859
+	+	+	+		+		+	12	158.2408	-290.739	48.96772
	+	+	+			+	+	11	156.7781	-290.09	49.61672
	+		+			+		9	154.5334	-290.078	49.62848
+	+	+			+	+		11	156.4374	-289.408	50.29812
	+	+		+		+	+	11	156.3937	-289.321	50.38552
	+	+		+	+	+		11	156.3192	-289.172	50.53468
	+	+	+			+		10	154.9793	-288.743	50.9633
+	+	+			+	+	+	12	156.8192	-287.895	51.81087
+		+			+	+		8	152.2931	-287.799	51.90701
	+	+		+	+	+	+	12	156.5062	-287.269	52.43701
+	+	+		+				10	154.2077	-287.2	52.50631

woody	dispersal	soil1_simp	density	maxD	log (seedwt)	log $(seedwt)^2$	meanlat	df	logLik	AICc	delta
	+	+	+	+	+		+	12	155.8936	-286.044	53.66207
+		+			+	+	+	9	152.4184	-285.848	53.85853
+	+	+		+			+	11	154.3439	-285.221	54.48512
+	+	+	+	+				11	154.226	-284.985	54.72101
	+	+	+		+		+	11	154.1819	-284.897	54.80928
	+	+		+				9	151.6541	-284.319	55.38714
	+	+		+			+	10	152.5473	-283.879	55.82725
	+	+		+	+		+	11	153.5051	-283.544	56.16282
+	+			+		+	+	11	153.4777	-283.489	56.21765
+	+			+		+		10	152.3348	-283.454	56.25211
+	+	+	+	+			+	12	154.3442	-282.945	56.76104
	+	+		+	+			10	151.9921	-282.769	56.93766
	+	+	+	+			+	11	152.9062	-282.346	57.3605
	+	+	+	+				10	151.7738	-282.332	57.37418
	+		+		+		+	10	151.5447	-281.874	57.83241
	+			+		+		9	150.4125	-281.836	57.87029
	+	+	+	+	+			11	152.5413	-281.616	58.09038
+	+		+		+		+	11	152.531	-281.595	58.11097
	+			+	+	+		10	151.1358	-281.056	58.65021
	+		+	+	+		+	11	152.2062	-280.946	58.76062
+	+		+	+	+		+	12	153.2154	-280.688	59.01864
	+			+		+	+	10	150.4195	-279.624	60.0828
+		+	+		+		+	9	149.1146	-279.24	60.46606
	+			+	+	+	+	11	151.2024	-278.938	60.76815
+		+	+	+	+		+	10	149.9911	-278.767	60.93965
+		+	+	+	+			9	148.6376	-278.286	61.42019
+	+	+	+		+			11	150.5505	-277.634	62.07204
+		+		+	+			8	146.8488	-276.911	62.79557
+	+				+	+		10	148.6891	-276.163	63.54352
+		+		+	+		+	9	147.3696	-275.75	63.95607
+	+		+	+	+			11	149.1133	-274.76	64.9464
+				+	+	+		8	145.6204	-274.454	65.25232

woody	dispersal	soil1_simp	density	maxD	log (seedwt)	log $(seedwt)^2$	meanlat	df	logLik	AICc	delta
+				+	+	+	+	9	146.6834	-274.378	65.32855
+	+				+	+	+	11	148.6898	-273.913	65.79339
+	+	+			+		+	11	148.3173	-273.168	66.53848
	+		+	+	+			10	147.1625	-273.109	66.59687
+		+	+		+			8	144.7809	-272.775	66.93137
+	+	+				+		10	146.3978	-271.58	68.1261
			+	+	+	+	+	9	145.2266	-271.464	68.2422
+					+	+		7	142.8359	-271.063	68.64319
	+	+	+				+	10	145.9481	-270.681	69.02568
			+		+	+	+	8	143.3253	-269.864	69.84271
+	+	+				+	+	11	146.535	-269.603	70.10291
		+	+	+	+	+	+	10	145.2796	-269.344	70.36262
	+		+	+			+	10	145.1751	-269.135	70.57156
+					+	+	+	8	142.8787	-268.971	70.73578
	+			+	+		+	10	145.0772	-268.939	70.76748
	+	+			+	+	+	11	146.0272	-268.588	71.11852
	+		+	+				9	143.7731	-268.557	71.14916
+			+		+		+	8	142.618	-268.449	71.25723
+	+	+	+				+	11	145.9544	-268.442	71.2642
	+			+	+			9	143.6601	-268.331	71.37523
			+		+	+		7	141.4444	-268.28	71.4262
+		+			+		+	8	142.5245	-268.262	71.44413
	+	+	+		+			10	144.7131	-268.211	71.49565
		+	+		+	+	+	9	143.4137	-267.838	71.868
+	+			+	+			10	144.475	-267.735	71.97182
+	+		+	+			+	11	145.5808	-267.695	72.01135
	+			+				8	142.2192	-267.651	72.05484
+	+			+	+		+	11	145.4754	-267.484	72.22228
+	+		+		+			10	144.1553	-267.095	72.61114
			+	+	+	+		8	141.8898	-266.993	72.71353
	+	+			+		+	10	143.9973	-266.779	72.9271
	+			+			+	9	142.8185	-266.648	73.0583

woody	dispersal	soil1_simp	density	maxD	log (seedwt)	log $(\text{seedwt})^2$	meanlat	df	logLik	AICc	delta
+	+		+	+				10	143.7882	-266.361	73.34539
+			+	+	+		+	9	142.6281	-266.267	73.43913
		+	+		+	+		8	141.4444	-266.102	73.60432
	+	+				+	+	10	143.3926	-265.57	74.1366
+	+			+				9	142.22	-265.451	74.25542
		+	+	+	+	+		9	141.9774	-264.966	74.74057
	+		+		+			9	141.9067	-264.824	74.88196
+	+			+			+	10	142.9229	-264.63	75.07603
	+	+				+		9	141.55	-264.111	75.59539
	+	+	+					9	141.4845	-263.98	75.72637
	+	+			+	+		10	142.5882	-263.961	75.74543
	+		+				+	9	141.3205	-263.652	76.05435
+			+	+	+			8	140.1094	-263.432	76.27434
	+				+	+	+	10	142.2491	-263.283	76.42369
+	+		+				+	10	142.1928	-263.17	76.53626
+	+	+	+					10	142.1808	-263.146	76.56024
+			+		+			7	138.6968	-262.785	76.92136
		+	+		+		+	8	139.5655	-262.344	77.36216
+	+					+		9	140.6029	-262.217	77.48947
	+				+	+		9	140.419	-261.849	77.85728
			+		+		+	7	138.1462	-261.684	78.02253
	+	+					+	9	140.0639	-261.139	78.56756
		+	+	+	+		+	9	139.6751	-260.361	79.34511
+	+					+	+	10	140.6167	-260.018	79.68845
			+	+	+		+	8	138.3839	-259.981	79.72534
	+					+	+	9	139.4548	-259.921	79.78578
+	+	+			+			10	140.4331	-259.651	80.05561
+	+	+					+	10	140.3509	-259.486	80.21999
+		+			+			7	136.7376	-258.867	80.83979
	+		+					8	137.0765	-257.366	82.34016
+	+		+					9	137.1034	-255.218	84.48845
			+		+			6	133.1045	-253.755	85.95131

woody	dispersal	soil1_simp	density	maxD	log (seedwt)	log $(seedwt)^2$	meanlat	df	logLik	AICc	delta
		+	+		+			7	134.1544	-253.7	86.00618
+	+	+						9	136.2448	-253.501	86.20564
+				+	+			7	134.0356	-253.463	86.2438
		+	+	+	+			8	134.9237	-253.06	86.64588
	+				+		+	9	135.8943	-252.8	86.90672
	+	+						8	134.7587	-252.73	86.97586
			+	+	+			7	133.6255	-252.642	87.06402
+				+	+		+	8	134.573	-252.359	87.34719
	+	+			+			9	135.5413	-252.094	87.61277
		+		+	+		+	8	133.5515	-250.316	89.39031
		+			+		+	7	132.0664	-249.524	90.18217
		+		+	+	+	+	9	134.0008	-249.013	90.69379
		+			+	+	+	8	132.6985	-248.61	91.0963
		+		+	+	+		8	132.5252	-248.264	91.44281
		+		+	+			7	131.171	-247.733	91.97301
				+	+	+		7	130.8895	-247.17	92.53598
+					+		+	7	130.8472	-247.086	92.6207
	+						+	8	131.894	-247.001	92.70513
				+	+	+	+	8	131.6623	-246.538	93.16852
					+	+	+	7	130.4044	-246.2	93.50621
+	+						+	9	132.4209	-245.853	93.85349
				+	+		+	7	129.894	-245.179	94.52698
					+	+		6	128.3936	-244.333	95.37325
		+			+	+		7	129.4275	-244.246	95.45999
				+	+			6	128.1474	-243.841	95.86559
					+		+	6	128.064	-243.674	96.03238
		+	+				+	7	128.4209	-242.233	97.47314
		+	+					6	127.2579	-242.062	97.64464
		+	+	+				7	128.1972	-241.786	97.92053
		+	+	+			+	8	128.8592	-240.932	98.77478
	+				+			8	128.8196	-240.852	98.85396
		+	+			+	+	8	128.7735	-240.76	98.94619

woody	dispersal	soil1_simp	density	maxD	log (seedwt)	log $(seedwt)^2$	meanlat	df	logLik	AICc	delta
+					+			6	126.5263	-240.599	99.10779
	+							7	127.5968	-240.585	99.1214
+		+	+	+				8	128.4898	-240.193	99.51359
		+		+				6	126.3114	-240.169	99.53763
+		+	+					7	127.3775	-240.146	99.55994
+		+	+				+	8	128.4281	-240.069	99.63709
		+	+			+		7	127.2581	-239.908	99.7988
		+	+	+		+		8	128.2696	-239.752	99.95407
		+	+	+		+	+	9	129.331	-239.673	100.0334
+	+				+			9	129.3173	-239.646	100.0608
		+			+			6	125.9831	-239.512	100.1942
+		+		+				7	127.033	-239.457	100.249
			+					5	124.6454	-238.968	100.738
			+				+	6	125.642	-238.83	100.8764
		+		+			+	7	126.7035	-238.798	100.908
+		+	+	+			+	9	128.8919	-238.795	100.9115
+		+		+		+		8	127.7466	-238.706	100.9999
		+		+		+		7	126.5895	-238.57	101.136
+		+	+			+	+	9	128.7736	-238.558	101.1482
+		+	+	+		+		9	128.7635	-238.538	101.1683
+	+							8	127.5982	-238.41	101.2968
		+		+		+	+	8	127.5341	-238.281	101.4251
+			+				+	7	126.3591	-238.11	101.5968
			+	+				6	125.279	-238.104	101.6024
+		+	+			+		8	127.3883	-237.99	101.7167
+		+	+	+		+	+	10	129.454	-237.693	102.0138
+		+		+			+	8	127.1303	-237.474	102.2327
+		+		+		+	+	9	128.1878	-237.387	102.3198
+			+					6	124.8454	-237.237	102.4695
			+	+			+	7	125.883	-237.157	102.549
			+			+		6	124.7109	-236.968	102.7385
			+			+	+	7	125.6904	-236.772	102.9342

woody	dispersal	soil1_simp	density	maxD	log (seedwt)	log (seedwt)^2	meanlat	df	logLik	AICc	delta
+			+	+				7	125.3989	-236.189	103.5173
+			+	+			+	8	126.4206	-236.054	103.652
+			+			+	+	8	126.3918	-235.997	103.7095
			+	+		+		7	125.2818	-235.955	103.7515
					+			5	123.0166	-235.711	103.9957
+			+			+		7	124.9728	-235.337	104.3694
			+	+		+	+	8	125.959	-235.131	104.5753
+			+	+		+		8	125.4191	-234.051	105.6551
+			+	+		+	+	9	126.466	-233.943	105.7634
		+					+	6	123.1452	-233.836	105.87
				+				5	122.0458	-233.769	105.9372
		+				+	+	7	123.9868	-233.365	106.3414
				+			+	6	122.2507	-232.047	107.659
		+						5	121.0878	-231.853	107.8534
				+		+		6	122.0768	-231.7	108.0067
+		+					+	7	123.148	-231.687	108.019
+				+				6	122.0701	-231.686	108.0203
+		+				+	+	8	124.0152	-231.244	108.4628
+		+						6	121.3954	-230.337	109.3695
				+		+	+	7	122.4049	-230.201	109.5053
+				+			+	7	122.3706	-230.132	109.5739
		+				+		6	121.0883	-229.723	109.9838
+				+		+		7	122.0918	-229.575	110.1314
+		+				+		7	121.4359	-228.263	111.4433
+				+		+	+	8	122.5108	-228.235	111.4717
							+	5	118.1236	-225.925	113.7817
+							+	6	119.062	-225.67	114.0363
								4	116.4444	-224.675	115.0313
						+	+	6	118.201	-223.948	115.7584
+						+	+	7	119.1398	-223.671	116.0354
+								5	116.7407	-223.159	116.5475
						+		5	116.5875	-222.852	116.8539

woody	dispersal	soil1_simp	density	maxD	log (seedwt)	log $(seedwt)^2$	meanlat	df	logLik	AICc	delta
+						+		6	116.9601	-221.466	118.2402
	+					+		8	NA	NA	NA
+	+				+		+	10	NA	NA	NA
+	+	+	+		+	+	+	13	NA	NA	NA

Table S6: Model selection results for the glmm with independent TPR (TPR_mpa) as the response variable, showing the ranking of all models as determined by the 'dredge' function. Predictor variables are listed in the first 8 columns, with "+" indicating that the predictor was included in the model. See table S3 for explanation of predictor variable codes. df = degrees of freedom, logLik = log-likelihood.

woody	dispersal	soil1_simp	density	maxD	log(seedwt)	meanlat	df	logLik	AICc	delta
+	+	+	+	+	+	+	12	-1158.39	2342.527	0
+	+		+	+	+	+	11	-1161.08	2345.629	3.101994
+	+		+		+	+	10	-1166.25	2353.709	11.18187
+	+	+	+		+	+	11	-1166.15	2355.776	13.24935
+	+		+			+	9	-1172.12	2363.236	20.70855
+	+	+	+	+		+	11	-1170.54	2364.543	22.01609
+	+		+	+		+	10	-1171.72	2364.656	22.12909
+	+	+	+			+	10	-1171.74	2364.697	22.16974
+	+		+	+	+		10	-1220.29	2461.789	119.2623
+	+	+	+	+	+		11	-1220.21	2463.896	121.3689
+	+		+	+			9	-1223.32	2465.638	123.111
+	+	+	+	+			10	-1223.01	2467.226	124.699
+	+	+		+	+	+	11	-1232.5	2488.476	145.9488
+	+	+		+		+	10	-1235.43	2492.083	149.556
	+	+	+	+	+	+	11	-1236.19	2495.846	153.3186
	+	+	+		+	+	10	-1239.02	2499.247	156.72
+	+			+	+	+	10	-1242.2	2505.619	163.0915
	+		+	+	+	+	10	-1243.33	2507.884	165.3572
+	+			+		+	9	-1247.21	2513.408	170.8811
+	+	+				+	9	-1248.29	2515.56	173.0333
+	+	+			+	+	10	-1247.93	2517.082	174.5546
+	+	+		+	+		10	-1249.85	2520.915	178.3876
+	+	+		+			9	-1252.61	2524.205	181.6782
+	+				+	+	9	-1252.81	2524.61	182.0833
+	+					+	8	-1254.35	2525.49	182.9627
	+		+		+	+	9	-1253.94	2526.872	184.3454
+	+			+	+		9	-1259.61	2538.214	195.6869
+	+			+			8	-1264.01	2544.804	202.2773

woody	dispersal	soil1_simp	density	maxD	log(seedwt)	meanlat	df	logLik	AICc	delta
+	+	+	+		+		10	-1267.2	2555.61	213.0825
	+	+	+	+	+		10	-1274.19	2569.604	227.0769
	+		+	+	+		9	-1285.96	2590.911	248.3844
	+	+		+	+	+	10	-1288.31	2597.841	255.314
+	+		+		+		9	-1291.26	2601.516	258.9885
	+	+		+	+		9	-1299.01	2617.015	274.4882
	+	+			+	+	9	-1303.88	2626.751	284.224
	+	+	+		+		9	-1314.46	2647.914	305.3866
	+		+		+		8	-1316.19	2649.164	306.6368
+	+	+	+				9	-1324	2666.985	324.4582
	+	+		+		+	9	-1324.75	2668.482	325.9551
	+	+	+	+		+	10	-1324.55	2670.32	327.7931
+		+	+	+	+	+	9	-1326.38	2671.756	329.2294
	+	+		+			8	-1330.86	2678.507	335.9805
	+	+	+	+			9	-1329.89	2678.774	336.247
+			+	+	+	+	8	-1333.96	2684.709	342.1821
+		+	+	+		+	8	-1338.46	2693.7	351.1726
	+			+	+	+	9	-1339.18	2697.349	354.8218
	+				+	+	8	-1345.12	2707.034	364.5072
	+			+	+		8	-1345.58	2707.938	365.4114
+			+	+		+	7	-1349.63	2713.872	371.3448
+			+		+	+	7	-1357.71	2730.032	387.5051
+		+	+		+	+	8	-1357.05	2730.896	388.3689
+		+	+			+	7	-1364.97	2744.551	402.0238
+			+			+	6	-1366.79	2746.043	403.516
	+	+				+	8	-1381.31	2779.414	436.8871
	+	+	+			+	9	-1380.27	2779.533	437.0063
+	+	+			+		9	-1388.25	2795.499	452.9717
+	+		+				8	-1396.28	2809.349	466.8218
+	+				+		8	-1396.51	2809.81	467.2828
+	+	+					8	-1402.31	2821.416	478.8885
		+	+	+	+	+	8	-1410.6	2837.985	495.458

woody	dispersal	soil1_simp	density	maxD	log(seedwt)	meanlat	df	logLik	AICc	delta
+		+	+	+	+		8	-1411.1	2838.98	496.4529
		+	+		+	+	7	-1414.06	2842.723	500.1956
	+			+		+	8	-1416.05	2848.889	506.3624
+			+	+	+		7	-1417.37	2849.344	506.8175
	+			+			7	-1417.93	2850.46	507.9333
	+		+	+		+	9	-1415.98	2850.954	508.4268
	+		+	+			8	-1417.92	2852.618	510.0907
			+	+	+	+	7	-1423.19	2860.98	518.4534
	+				+		7	-1424.57	2863.739	521.2118
	+	+			+		8	-1424.19	2865.172	522.6452
+		+	+		+		7	-1425.67	2865.956	523.4285
+		+	+	+			7	-1426.24	2867.097	524.5696
			+		+	+	6	-1428.3	2869.052	526.5249
+		+				+	6	-1430.32	2873.095	530.568
+		+		+		+	7	-1429.95	2874.515	531.9882
+		+			+	+	7	-1430.3	2875.213	532.686
+						+	5	-1432.49	2875.3	532.7726
+				+		+	6	-1431.7	2875.847	533.3202
+		+		+	+	+	8	-1429.93	2876.649	534.122
+					+	+	6	-1432.38	2877.206	534.6787
+				+	+	+	7	-1431.62	2877.856	535.3293
+	+						7	-1433.04	2880.683	538.1555
+			+	+			6	-1434.33	2881.122	538.5952
+		+	+				6	-1450.88	2914.224	571.6968
	+		+			+	8	-1450.55	2917.884	575.3566
	+					+	7	-1452.89	2920.388	577.8606
		+	+	+	+		7	-1454.45	2923.514	580.9869
			+	+	+		6	-1458.45	2929.35	586.8226
		+			+	+	6	-1458.73	2929.905	587.3775
		+		+	+	+	7	-1458.23	2931.062	588.5346
+				+			5	-1466.33	2942.984	600.4567
+				+	+		6	-1465.64	2943.73	601.2027

woody	dispersal	soil1_simp	density	maxD	log(seedwt)	meanlat	df	logLik	AICc	delta
+		+		+			6	-1466.3	2945.05	602.5228
+		+		+	+		7	-1465.44	2945.481	602.9537
+			+		+		6	-1469.32	2951.089	608.5616
					+	+	5	-1482.74	2975.811	633.2842
				+	+	+	6	-1482.49	2977.427	634.8999
		+		+	+		6	-1484.32	2981.103	638.576
		+	+		+		6	-1494.82	3002.098	659.5715
				+	+		5	-1496.97	3004.27	661.7431
			+		+		5	-1500.1	3010.515	667.9875
		+		+		+	6	-1503.77	3020.001	677.4743
		+	+	+		+	7	-1503.77	3022.153	679.6264
		+				+	5	-1514.25	3038.827	696.3001
		+	+			+	6	-1514.14	3040.729	698.2019
+		+			+		6	-1514.23	3040.91	698.3831
+		+					5	-1518.04	3046.409	703.8816
+			+				5	-1524.59	3059.511	716.9845
		+	+	+			6	-1528.49	3069.442	726.9146
		+		+			5	-1529.69	3069.695	727.1676
+					+		5	-1533.03	3076.382	733.8547
				+		+	5	-1547.89	3106.093	763.566
			+	+		+	6	-1547.86	3108.173	765.6463
+							4	-1551.78	3111.765	769.2382
		+			+		5	-1552.26	3114.834	772.3072
					+		4	-1553.59	3115.396	772.8686
						+	4	-1557.43	3123.071	780.5436
			+			+	5	-1557.37	3125.061	782.5344
				+			4	-1560.16	3128.541	786.0138
			+	+			5	-1559.15	3128.622	786.0954
	+		+				7	-1635.09	3284.779	942.2524
	+						6	-1636.2	3284.854	942.327
	+	+					7	-1636.13	3286.871	944.3437
	+	+	+				8	-1635.07	3286.934	944.4069

woody	dispersal	soil1_simp	density	maxD	log(seedwt)	meanlat	df	logLik	AICc	delta
			+				4	-1702.69	3413.598	1071.071
		+	+				5	-1702.37	3415.059	1072.532
							3	-1710.34	3426.812	1084.285
		+					4	-1709.62	3427.454	1084.927

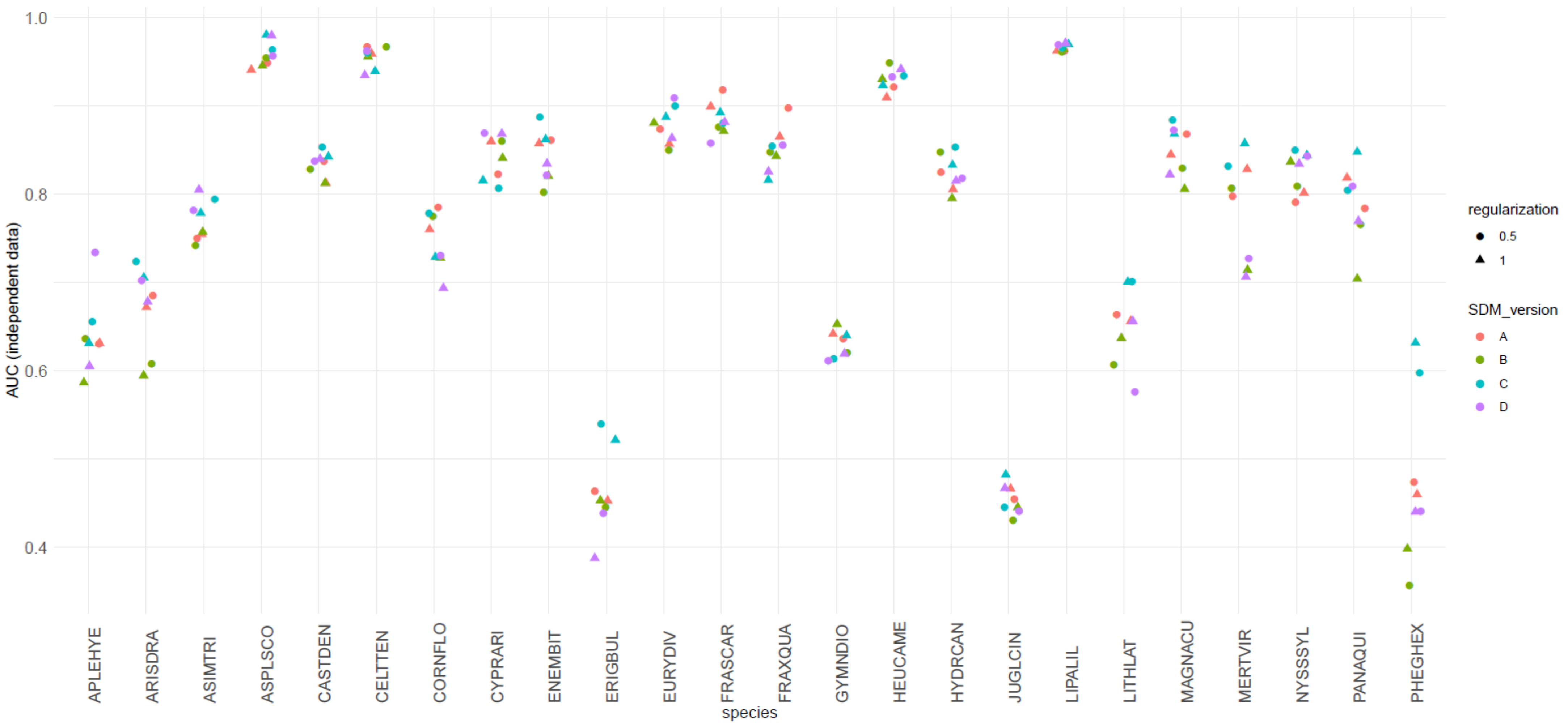

Figure S1: AUC based on independent presences and absences for each of the eight SDM versions for each species. Regularization was set to either 0.5 or 1. SDM_version refers to which set of predictors were used (see Table S2 above). Species codes are the first four letters of the genus followed by the first three letters of the species name. See Table S4 for full scientific names.

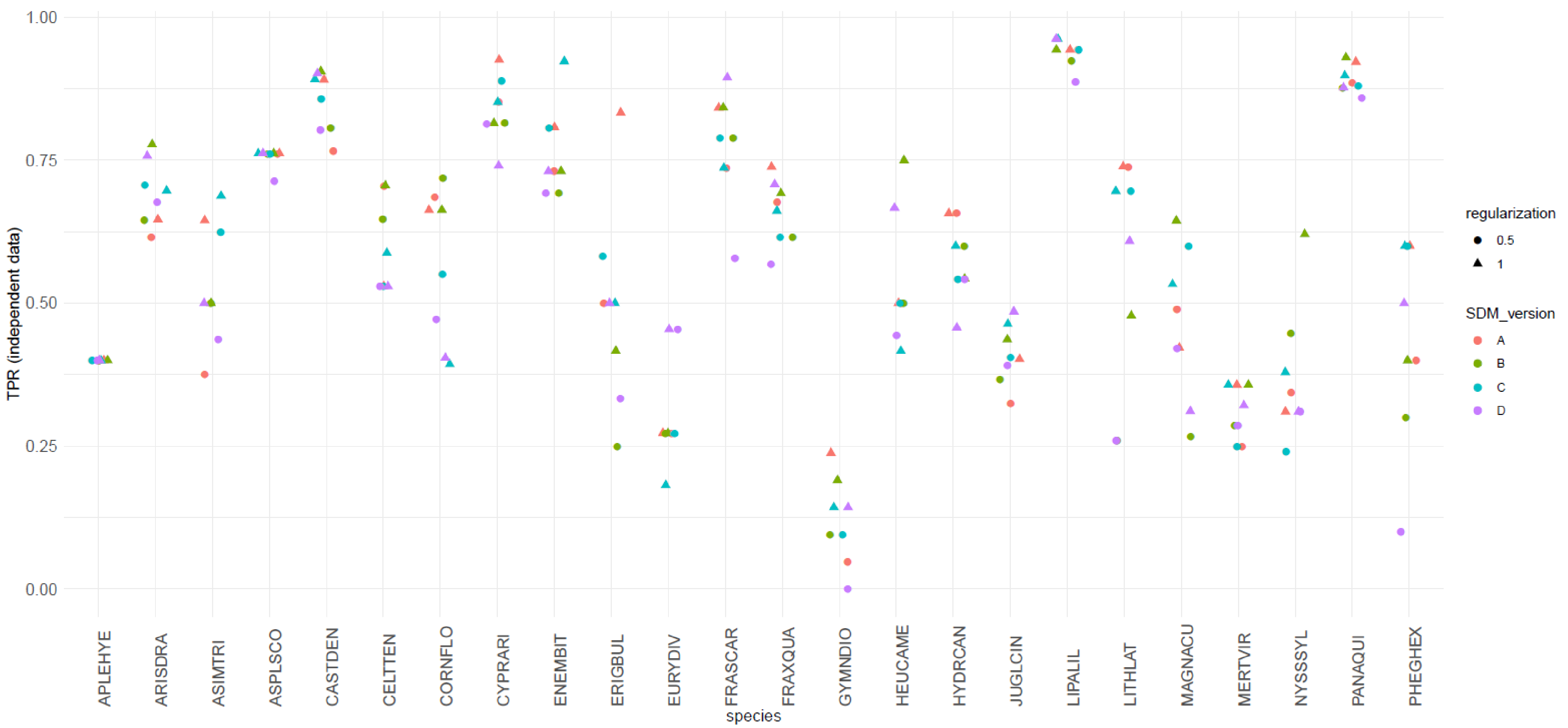

Figure S2: TPR (true positive rate) based on independent presences and absences for each of the eight SDM versions for each species. Regularization was set to either 0.5 or 1. SDM_version refers to which set of predictors were used (see Table S2 above). Species codes are the first four letters of the genus followed by the first three letters of the species name. See Table S4 for full scientific names.

References

McKenney, D. W., M. F. Hutchinson, P. Papadopol, K. Lawrence, J. Pedlar, K. Campbell, E. Milewska, R. F. Hopkinson, D. Price, and T. Owen. 2011. Customized spatial climate models for North America. Bulletin of the American Meteorological Society, December: 1611-1622.

Williams, J. N., C. Seo, J. Thorne, J. K. Nelson, S. Erwin, J. M. O'Brien, and M. W. Schwartz. 2009. Using species distribution models to predict new occurrences for rare plants. Diversity and Distributions 15:565-576.
